# FAK modulates glioblastoma stem cell energetics via regulation of glycolysis and glutamine oxidation

**DOI:** 10.1242/dmm.052634

**Published:** 2025-11-28

**Authors:** Roza H. A. Masalmeh, John C. Dawson, Virginia Alvarez Garcia, Morwenna T. Muir, Roderick N. Carter, Giles E. Hardingham, Cameron Davies, Rosina Graham, Alex von Kriegsheim, Jair Marques, Chinmayi Pednekar, Steven M. Pollard, Neil O. Carragher, Valerie G. Brunton, Margaret C. Frame

**Affiliations:** ^1^ Cancer Research UK Scotland Centre (Edinburgh), Institute of Genetics and Cancer, University of Edinburgh, Crewe Road South, Edinburgh EH4 2XU, UK; ^2^ Department of Physiology and Pharmacology, School of Medicine, University of Cantabria and Instituto de Investigacion Valdecilla (IDIVAL), 39011 Santander, Spain; ^3^ UK Dementia Research Institute, Chancellor's Building, University of Edinburgh, 49 Little France Crescent, Edinburgh EH16 4SB, UK; ^4^ Simons Initiative for the Developing Brain, University of Edinburgh, Edinburgh EH8 9XF, UK; ^5^ Centre for Discovery Brain Sciences, Hugh Robson Building, University of Edinburgh, Edinburgh EH8 9XD, UK; ^6^ Centre for Regenerative Medicine, Institute for Regeneration and Repair, University of Edinburgh, 5 Little France Drive, Edinburgh EH16 4UU, UK

**Keywords:** Adhesion proteins, Glycolysis, Glutamine oxidation, Mitochondria, Mechanical forces, Extracellular matrix

## Abstract

Glycolysis and the tricarboxylic acid cycle (TCA) cycle are reprogrammed in cancer cells to meet bioenergetic and biosynthetic demands, including by engagement with the extracellular matrix (ECM). However, the mechanisms by which the ECM engagement reprograms core energy metabolism is still unknown. We showed that the canonical cell−ECM adhesion protein focal adhesion kinase (FAK, also known as PTK2) and, specifically, its kinase activity, is driving cellular energetics. Using a mouse stem cell model of glioblastoma, we showed that deletion of the FAK gene simultaneously inhibits glycolysis and glutamine oxidation, increases mitochondrial fragmentation, elevates phosphorylation of the mitochondrial protein MTFR1L at serine residue 235 (S235) and triggers a mesenchymal-to-epithelial transition. These metabolic and structural changes arise through altered contractility of actomyosin, as shown by myosin light chain type II (MYL2, also known as MLC2) phosphorylated (p) at S19. This process can be reversed by Rho-kinase (ROCK) inhibitors revealing mechanotransduction pathway control of both mitochondrial dynamics and glutamine oxidation. FAK-dependent metabolic programming is associated with regulation of cell migration, invasive capacity and tumour growth *in vivo*. Our work describes a previously unrecognised FAK–ROCK axis that couples mechanical cues to the rewiring of energy metabolism, linking cell shape, mitochondrial function and malignant behaviour.

## Teaser

**Summary:** The key adhesion protein focal adhesion kinase (FAK) transduces adhesion signals to enhance glycolysis and oxidative phosphorylation in a glioblastoma stem-cell model, highlighting a targetable bioenergetic dependency.

## INTRODUCTION

Glioblastoma (GBM) is the most common and lethal form of malignant primary brain tumour (Noroxe et al., 2016). The standard treatment for this cancer involves surgical resection, radiation therapy and chemotherapy. However, none of these regimens are curative. One of the reasons why GBM is resistant to therapy is the presence of a subset of cells within the tumour with high tumorigenic capacity, termed GBM stem cells (Osuka and Van Meir, 2017; Singh et al., 2004). These cells can drive recurrence and heterogeneity by rewiring their signalling and metabolism, thereby adapting to restricted nutrition and overcoming therapies (Renoult et al., 2024). The ability of cancer cells to reprogram energy metabolism has emerged as a hallmark of cancer, and the ability of cancer cells to shift between different metabolic states has been recently recognized as a therapeutic target (Pelaz et al., 2017; Hanahan and Weinberg, 2011). A recent study, investigating biological traits of human GBM tumours, revealed at least four tumour cell states, i.e. proliferative/progenitor, neuronal, mitochondrial and glycolytic/plurimetabolic (Garofano et al., 2021). GBM stem cells classified in the mitochondrial state critically depend on oxidative phosphorylation (OXPHOS) to produce energy, whereas the glycolytic/plurimetabolic subtype utilises multiple energy-producing pathways, e.g. glycolysis, amino acid and lipid metabolism, thereby granting cancer cells metabolic adaptability and protection from oxidative stress and cell death. Amongst the four subtypes, the mitochondrial subtype has significantly better clinical outcome and is more susceptible to OXPHOS inhibitors (Garofano et al., 2021). This highlights the importance of understanding metabolic pathways in GBM and the potential for finding metabolic vulnerabilities that we can exploit therapeutically.

One of the ways by which metabolism is altered in cancers is through responding to cues from the tumour microenvironment. For example, increasing extracellular matrix (ECM) stiffness can modulate glycolysis, increase the activity of metabolic enzymes, and affect mitochondrial morphology and function (Tharp et al., 2021; Park et al., 2020; Papalazarou et al., 2020). These alterations help cancer cells resist oxidative stress and promote the development of invasive and metastatic phenotypes. The actin cytoskeleton can function as a scaffold for some glycolytic enzymes that can modulate glycolysis through spatial and functional regulation of these enzymes (DeWane et al., 2021). An unanswered question is whether, and how, this is connected to the role of dynamic actin remodelling in processes – such as cell migration, or hallmark changes in cell state – such as epithelial-to-mesenchymal transition (EMT). The links between cell−ECM mechanical cues, the actin cytoskeleton and metabolism are beginning to be identified; yet we do not know which integrin may contribute.

Focal adhesion kinase (FAK, officially known as PTK2) is a key signal transduction regulator found at integrin adhesions (Frame et al., 2010; Horton et al., 2015). Indeed, FAK is at the centre of one of the four focal adhesion nexuses that connect integrin complexes to actin and multiple intracellular signalling axes (Horton et al., 2015). FAK is also known to buffer adhesion and therapeutic stress in tumour cells and promote their survival (Dawson et al., 2021). In the context of GBM, integrin adhesion-related genes (e.g. *ITGB1* and *PTK2*) are essential genetic dependencies for the injury response transcriptional state as defined by Richards et al. and MacLeod et al. (Richards et al., 2021; MacLeod et al., 2024).

We set out to address the role of FAK in a transformed neural stem cell model of GBM as recently described (Gangoso et al., 2021; Loftus et al., 2024), and specifically addressed whether FAK can link the regulation of adhesion signalling to metabolic pathways and, if so, uncover the mechanisms. We found that FAK expression was increased upon oncogenic transformation and that CRISPR/Cas9-mediated genetic deletion of FAK reduced tumour growth *in vivo*. FAK deletion in these cells induced a profound change in both cell morphology and the actin cytoskeleton, along with concomitant changes in gene expression consistent with transition from a mesenchymal-like to an epithelial-like phenotype. As a result of these changes, cells were significantly less motile both in two-dimensional (2D) culture and three-dimensional (3D) invasion assays. Furthermore, loss of the gene encoding FAK significantly suppressed rates of both glycolysis and OXPHOS, rendering the cells energetically sluggish. This was associated with enhanced actomyosin contractility especially evident at cell−cell contacts and alterations in mitochondrial morphology. Treatment with multiple Rho-kinase (ROCK) inhibitors restored FAK-dependent mesenchymal morphology, low actomyosin contractility at cell–cell contacts, glutamine oxidation, and mitochondrial morphology. Therefore, FAK co-regulates actin, actomyosin contractility, migration and invasion via ROCK in a transformed neural stem cell GBM model, and we show here that this is closely linked to FAK-regulated mitochondrial morphology and cellular energetics. This describes a new cellular dependency regulated by FAK in GBM cells.

## RESULTS

### FAK controls GBM-associated cellular phenotypes

**Fig. 1.**
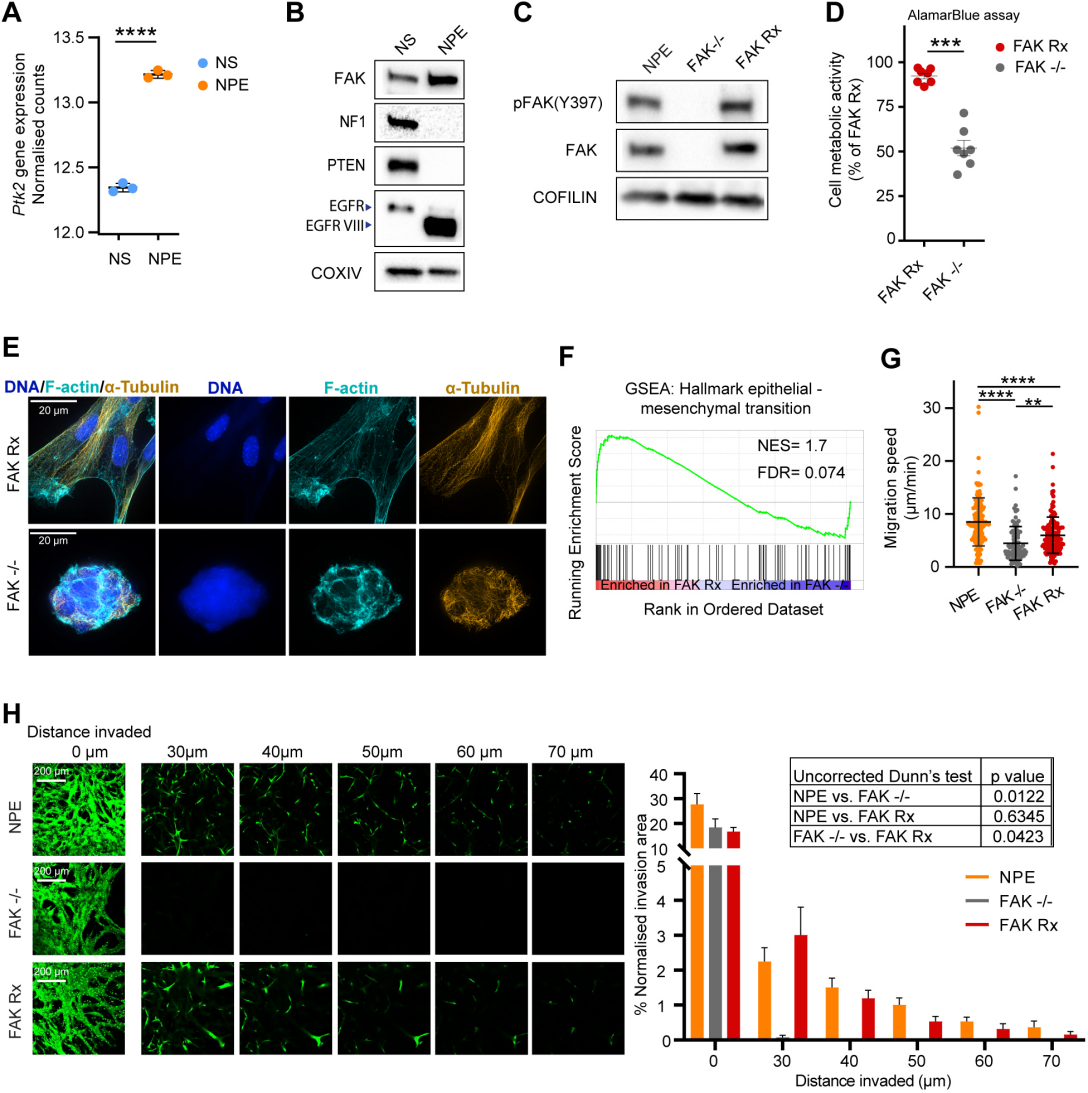
**Genetic deletion of FAK drives phenotypic and transcriptional changes in a GBM mouse stem cell model.** (A) Regularised-logarithm transformation (rlog) normalised gene expression counts of *Ptk2* in NS and NPE cells. Gene expression counts obtained from Gangoso et al., (2021) from (PMID: 33857425). Error bars indicate the mean ±s.d. Statistics: unpaired two-tailed *t*-test (*n*=3). ****P<0.001. (B) Western blot showing FAK, NF1, PTEN, EGFR and EGFRvIII protein levels in NS and NPE cells. COX IV was used as a loading control. The bands for EGFR and EGFRvIII are saturated in this exposure and are shown for qualitative purposes only. (C) Western blot analysis showing protein expression of pFAK(Y397) and FAK in NPE, FAK−/− and FAK Rx cells. Cofilin was used as a loading control. (D) Cell metabolic activity measured by alamarBlue assay of FAK Rx and FAK−/− cells cultured for 3 days at normal culture conditions. Error bars indicate the mean ±s.e.m. Statistics: unpaired two-tailed *t*-test (*n*=7). ***P<0.005. (E) Representative super-resolution microscopy images of FAK Rx and FAK−/− cells showing the F-actin cytoskeleton labelled with fluorophore-conjugated phalloidin (green) and α-tubulin (orange); nuclei were stained with DAPI (blue). Image brightness and contrast were autoscaled individually to enable visualisation. Scale bars: 20 µm. (F) Gene set enrichment analysis (GSEA) plot of the ‘Hallmark epithelial-mesenchymal transition’ gene set for proteins differentially regulated in FAK Rx cells compared to FAK−/− cells (*n*=3). Normalised enrichment score (NES) and false discovery rate (FDR) are shown. (G) Migration speed (in μm/min) of NPE, FAK−/− and FAK Rx cells plated on laminin I and imaged every 10 min for 48 h. Each dot represents one cell, data from two independent cultures. Error bars indicate the mean ±s.d. Statistics: Kruskal–Wallis test followed by Dunn's multiple comparison test. **P<0.01, ****P<0.001. (H) Left: Representative optical sections of NPE, FAK−/− and FAK Rx cells stained with Calcein AM invading through Matrigel. Scale bars: 200 μm. Right: Plotted is the percentage of cells present in each displayed optical section of total cell area throughout the Matrigel (*n*=3 technical repeats). Error bars indicate the mean +s.d. Statistics: Kruskal–Wallis test followed by non-adjusted Dunn's test.

The underlying mechanisms by which FAK may contribute to GBM are poorly understood. We examined the role of FAK in GBM by using a disease-relevant transformed mouse neural stem cell model that we have described previously (Gangoso et al., 2021; Loftus et al., 2024). The neural stem cell model is important because it is believed that neural stem cells are one of the potential cells of origin of GBM and that they give rise to GBM stem cells, which are thought to drive recurrence and therapeutic resistance (Bao et al., 2006). We used neural stem cells obtained from C57BL/6-SCRM mice, which had been modified to harbour co-deletion of the genes encoding neurofibromin 1 (*Nf1*) and phosphatase and tensin homolog (*Pten*), and that overexpress the most common extracellular domain variant of epidermal growth factor (EGFR), i.e. EGFRvIII [see review by Gan et al. (2013)]. We found that the mRNA levels of *Ptk2*, the mouse gene encoding FAK, is significantly increased in transformed NPE cells when compared to untransformed parental neural stem cells (*P*<0.0001) (Fig. 1A), and increased expression of the gene encoding FAK is paralleled by elevated FAK protein levels in NPE cells (Fig. 1B). CRISPR-Cas9-mediated targeting of the gene encoding FAK successfully generated several NPE clones devoid of FAK protein. We re-expressed FAK in the FAK-deficient (FAK−/−) clonal line no. 1 to levels equivalent to those in NPE cells, thus, generating the rescue cell line FAK-Rx. These isogenic FAK−/− and FAK-Rx cells only differ in the expression of FAK protein (Fig. 1C). To characterise the effects of deleting FAK on cellular phenotypes, we first assessed cell number, viability, and metabolic activity in FAK Rx and FAK−/− cells after three days in culture. While there was no significant difference in cell count or viability between the two groups, as determined by Trypan Blue exclusion (Fig. S1A,B), FAK−/− cells displayed a significant reduction in metabolic activity, as measured by the alamarBlue assay (Fig. 1D). These findings suggest that the decreased metabolic activity observed upon FAK deletion is not due to reduced proliferation. The loss of FAK also resulted in profound morphological changes. Cells expressing FAK were well attached to the cell culture substrate and displayed a spread and elongated mesenchymal-like morphology, while their comparable FAK−/− cells adopted a more rounded and less spread morphology. These FAK-deficient cells clustered tightly together with a more epithelial-like morphology and displayed reduced contact with the substratum. They were characterised by increased junctional F-actin at cell−cell contacts as a result of general remodelling of the actin and tubulin cytoskeletons (Fig. 1E). Notably, elongated morphology and increased cell spreading were observed at 48 h after nucleofecting the cells with the FAK-expressing vector, before commencing G418 selection to enrich for cells expressing FAK (Fig. S1C). These morphological changes were consistent across multiple FAK−/− clones (Fig. S1D), showing this was not due to any inter-clonal variability. The expression of FAK was associated with a significant enrichment of the gene set ‘Hallmark epithelial-mesenchymal transition’ (Howe et al., 2018) (FDR=0.074) (Fig. 1F and Fig. S1E). Consistent with these changes, the loss of FAK suppressed migration speed of NPE cells in 2D culture and reduced the distance of 3D invasion into Matrigel (Fig. 1G,H).

Deleting the gene encoding FAK in mouse embryonic fibroblasts results in increased expression of *Ptk2b* (also known and hereafter referred to as *Pyk2*). Therefore, we wanted to check if compensatory *Pyk2* expression is relevant to the observed phenotypes. We found that *Pyk2*/PYK2 are not detected at RNA/protein level in FAK−/− and FAK Rx cells (Fig. S1F), indicating that there is no compensatory increase in *Pyk2* expression when FAK is lost in these cells.

### FAK promotes glycolysis and glutamine oxidation in GBM mouse stem cells

**Fig. 2.**
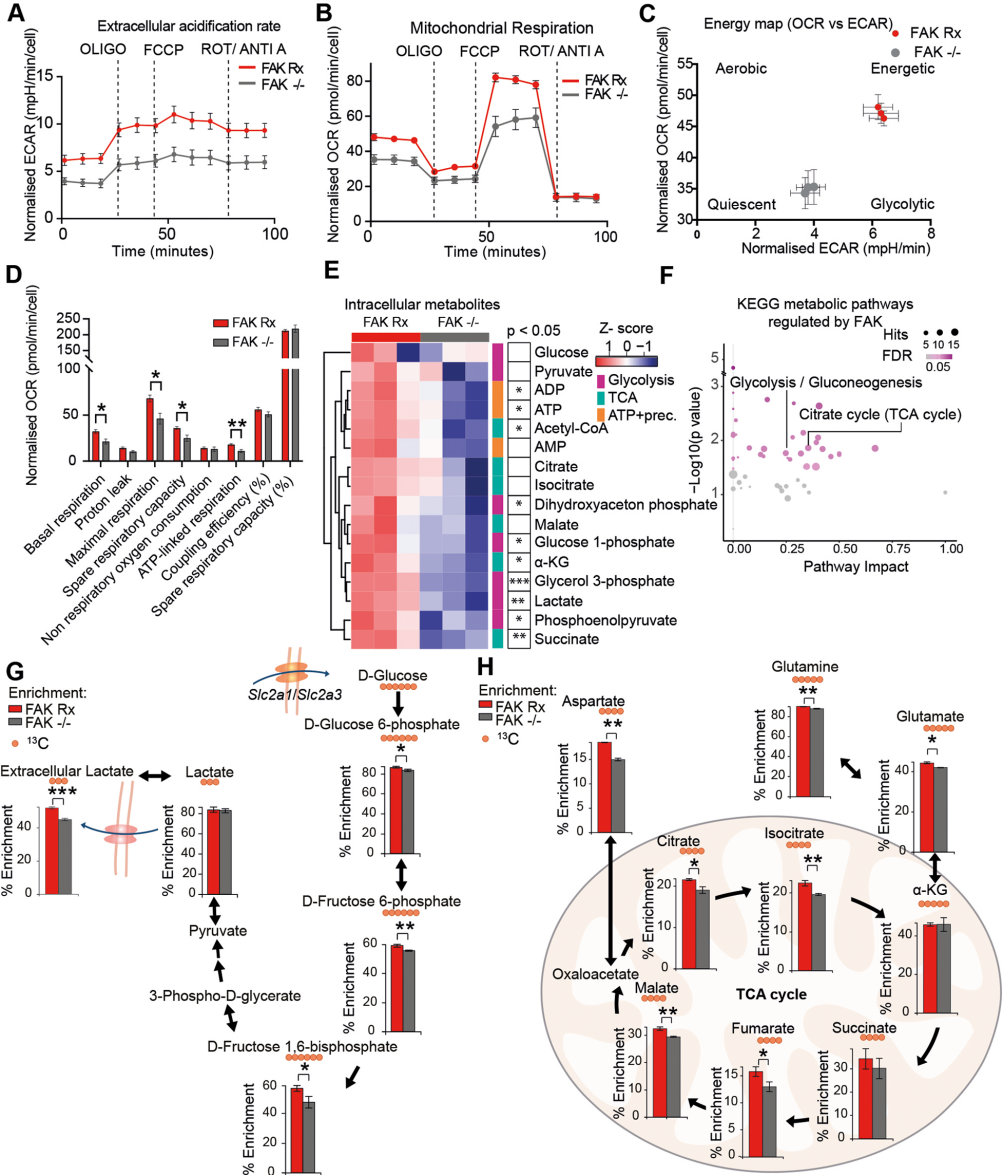
**FAK promotes glycolysis, mitochondrial respiration and glutamine oxidation.** (A,B) Extracellular acidification rate (ECAR) (A) and oxygen consumption rate (OCR) (B) of FAK Rx and FAK−/− cells (7000 cells per well, *n*=7 independent cultures, three replicate measures). Mitochondrial stress test conditions: basal, oligomycin (OLIGO), carbonyl cyanide-4 (trifluoromethoxy) phenylhydrazone (FCCP), a mixture of rotenone (ROT) and antimycin A (ANTI A). Error bars indicate the mean ±s.e.m. Dashed vertical lines indicate injections of oligomycin, FCCP and rotenone/antimycin A. (C) Basal ECAR vs OCR, calculated from data shown in A and B. Error bars indicate the mean ±s.e.m. (D) Bar graphs showing the quantification of mitochondrial parameters calculated from the normalized OCR data shown in B. Error bars indicate the mean ±s.e.m. Statistics: Mann–Whitney test. *P<0.05, **P<0.01. (E) Heatmap of intracellular metabolites as indicated. ATP+prec., ATP precursors. Shown is the normalised peak intensity in FAK Rx and FAK−/− cells. Each square represents one individual replicate (*n*=3 independent cultures per day). Statistics: unpaired two-tailed *t*-test. (F) Bubble plot showing glycolysis/glucogenesis and TCA cycle among the significantly enriched KEGG pathways in pathway enrichment analysis of intracellular metabolites in FAK Rx compared to FAK−/− cells. Circle sizes are proportional to the number of hits in that pathway. Darker colour represents more significance. (G,H) Atom fraction enrichment of glucose-derived ^13^C in glycolysis intermediates after incubation of FAK Rx and FAK−/− cells with ^13^C_6_ D-glucose for 1 h (G) or ^13^C_5_ glutamine for 3 h (H) (*n*=3 independent cultures on the same day). The main isotopologue of each metabolite is shown and plotted as the fraction of the sum of all isotopologues. Error bars indicate the mean±s.d. Statistics: unpaired two-tailed *t*-test. *P<0.05, **P<0.01, ***P<0.005.

In addition to the morphological and migratory phenotypes upon FAK modulation, we noticed that FAK Rx culture medium containing the pH indicator phenol red was consistently more acidic (as judged by development of a yellow colour) compared to that of FAK−/− culture medium. This led us to hypothesize that FAK may regulate cellular metabolism (Fig. S2A). To test this directly, we performed a Seahorse XF Cell Mito Stress assay and found that FAK loss reduced both the extracellular acidification rate (ECAR), a surrogate measure of glycolytic flux, and the oxygen consumption rate (OCR) (Fig. 2A,B). This suggests that FAK loss has dampened both of the two main catabolic pathways, i.e. glycolysis and mitochondrial OXPHOS. As a result, FAK−/− cells are energetically supressed and are tending towards quiescence (Fig. 2C). We found that spare respiration capacity, maximal respiration and respiration-linked ATP production were significantly reduced in FAK−/− cells when compared to FAK Rx cells (Fig. 2D). Consistent with this, cellular metabolite profiling revealed that FAK loss reduced glycolytic intermediates, tricarboxylic acid cycle (TCA) cycle metabolites, ATP and its precursors (Fig. 2E). In keeping with the observed metabolic effects, KEGG metabolic pathway enrichment analysis of 131 cellular metabolites supported the regulation of glycolysis, gluconeogenesis and TCA cycle in a FAK-dependent manner (Fig. 2F).

To further characterise the role of FAK in regulating glycolysis and the TCA cycle, we performed stable isotope-labelled ^13^C_6_-glucose tracing in FAK-expressing and FAK-deficient cells. We found significantly reduced labelling of glycolytic intermediates within cells and of secreted lactate, the end product of anaerobic glycolysis, in the absence of FAK (Fig. 2G). We also traced ^13^C_5_-glutamine and found reduced labelling in most TCA cycle metabolites in FAK−/− cells compared to FAK Rx cells (Fig. 2H), indicative of a role for FAK in efficient glutamine oxidation. Taken together, these findings have uncovered a previously unknown role for FAK in promoting both efficient glycolysis and glutamine oxidation.

We noted that the role of FAK in promoting glycolysis was consistently associated with higher levels of two glucose transporter proteins, namely glucose reporters 1 and 2 (GLUT1 and GLUT 3, respectively; officially known as SLC2A1 and SLC2A2, respectively) (Fig. S2B,C), although we have not addressed causality of these changes.

To supplement the FAK gene deletion approach, we also examined the effects of acute pharmacological inhibition of FAK kinase activity on glycolysis and glutamine oxidation. We treated NPE cells with the FAK inhibitor VS-4718 at a concentration of 300 nM. After 48 h of treatment, we observed a significant decrease (although not ablation) of FAK levels phosphorylated at tyrosine residue 397 [pFAK(Y397)] compared to those in vehicle-treated control cells (Fig. S2D). We found that the VS-4718 FAK inhibitor reduced glucose incorporation into glycolytic intermediates (Fig. S2E) as well as glutamine incorporation into TCA cycle intermediates (Fig. S2F). Taken together, our FAK gene deletion and FAK kinase inhibition experiments demonstrate a role for FAK, and FAK kinase activity, in optimal energy production via glycolysis and glutamine oxidation.

To also examine how suppressing FAK activity by impairing normal integrin signalling affects metabolism, we plated NPE cells on Poly-2-hydroxyethyl methacrylate (Poly 2-HEMA)-coated surfaces that do not support integrin signalling. We then assessed the effects on glutamine incorporation into the TCA cycle, comparing NPE cells on 0.075% Poly 2-HEMA surfaces to those plated on 1 µg/ml laminin. NPE cells on the Poly 2-HEMA-coated surface displayed decreased pFAK(Y397) levels as expected and reduced contact with the substratum compared to those on laminin (Fig. S3A,B). In keeping with our FAK gene deletion experiments, we found that suppression of signalling via FAK upon plating on Poly 2-HEMA caused reduced glutamine incorporation into TCA cycle intermediates (Fig. S3C).

### FAK regulates the length of mitochondria

**Fig. 3.**
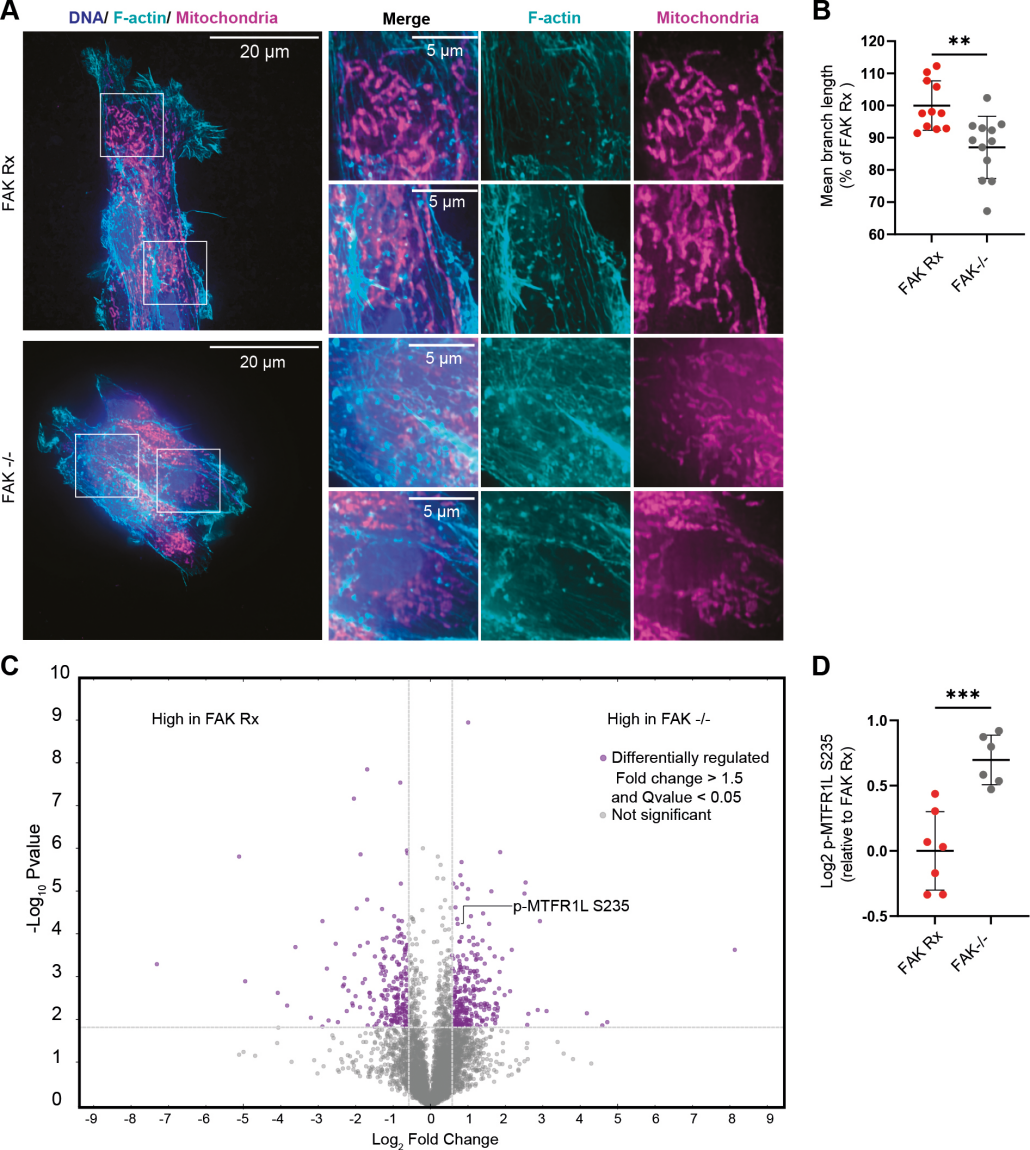
**FAK is associated with elongated mitochondria.** (A) Representative super-resolution microscopy images of FAK Rx and FAK−/− cells showing mitochondrial morphology stained with MitoTracker Deep Red FM (magenta) and F-actin cytoskeleton labelled with fluorophore-conjugated phalloidin (cyan); nuclei were stained with DAPI (blue). Boxed areas in main images are shown magnified on the right. Scale bars: 20 µm (full field of view), 5 µm (insets). (B) Quantification of mitochondrial mean fragment (branch) length in FAK Rx and FAK−/− cells. *n*=2 independent experiments. Each dot represents a field of view. Error bars indicate the mean ±s.d. Statistics: unpaired two-tailed *t*-test. **P<0.01. (C) Volcano plot of post-translational modifications highlighting the differentially modified proteins in purple. p-MTFR1L S235 is labelled. *n*=3 independent cultures. (D) Abundance of p-MTFR1L S235 in FAK Rx and FAK−/− cells. *n*=7 (FAK Rx) and *n*=6 (FAK−/−) independent cultures. Data from two separate experiments conducted on different days are shown. Error bars indicate the mean and ±s.e.m. Statistics: unpaired two-tailed *t*-test. ***P<0.005.

Previous studies have suggested that the shape of mitochondria is linked to their function and is responsive to nutrients and energy demands (reviewed in Chen et al., 2023). Specifically, it has been proposed that elongated mitochondria are associated with efficient OXPHOS (Yao et al., 2019; Baek et al., 2023; Wang et al., 2024). Given this, and the role of FAK in regulating cell adhesion and cytoskeletal structure in the NPE model of GBM used in our current study, we asked whether glutamine oxidation through FAK may be functionally linked to FAK-dependent control of mitochondrial morphology. We used super-resolution by optical pixel reassignment (SoRa) spinning disk microscopy to image the mitochondrial network stained with MitoTracker (Fig. 3A). We then used mitochondria features extraction tool (called Mitochondria Analyser Fiji/ImageJ plugin; Chaudhry et al., 2020; Schindelin et al., 2012) to define the mitochondrial network and extract different mitochondria features. This revealed that FAK loss decreased the mitochondria mean branch length (Fig. 3B), implying that FAK promotes elongated and filamentous mitochondria; this mitochondrial morphology was associated with more efficient OXPHOS (Fig. 2B,D,H). A comprehensive phosphoproteomic analysis of FAK-expressing and FAK-deficient cells revealed a number of changes, including that deletion of FAK leads to a significant increase in mitochondrial fission regulator 1 like (MTFR1L) phosphorylated at S235 (p-MTFR1L S235) (Fig. 3C,D). This phosphorylation of S235 by AMPK – a site that is orthologous to S238 in human MTFR1L – is known to result in mitochondrial fragmentation under metabolic stress (Tilokani et al., 2022) and is, therefore, a candidate mediator of the effects of FAK loss on mitochondrial morphology and GBM cellular energy production.

### FAK regulates length of mitochondria via ROCK

The shape and function of mitochondria is responsive to mechanical signals stimulated by interaction with the ECM (Tharp et al., 2021; Papalazarou et al., 2020). FAK is implicated in mechanotransduction – by connecting signals from the integrin family of ECM adhesion receptors to the actin cytoskeleton – and actomyosin contractility (Di et al., 2023); this has been shown to regulate the switch between epithelial-like and mesenchymal-like colon cancer cells (Avizienyte and Frame, 2005). Since the mesenchymal-like phenotype of the transformed NPE cells we used here was also FAK-dependent (Fig. 1E,F), we next addressed whether FAK regulates mechanotransduction in NPE cells and, if so, whether this is associated with changes in mitochondrial morphology and metabolism.

**Fig. 4.**
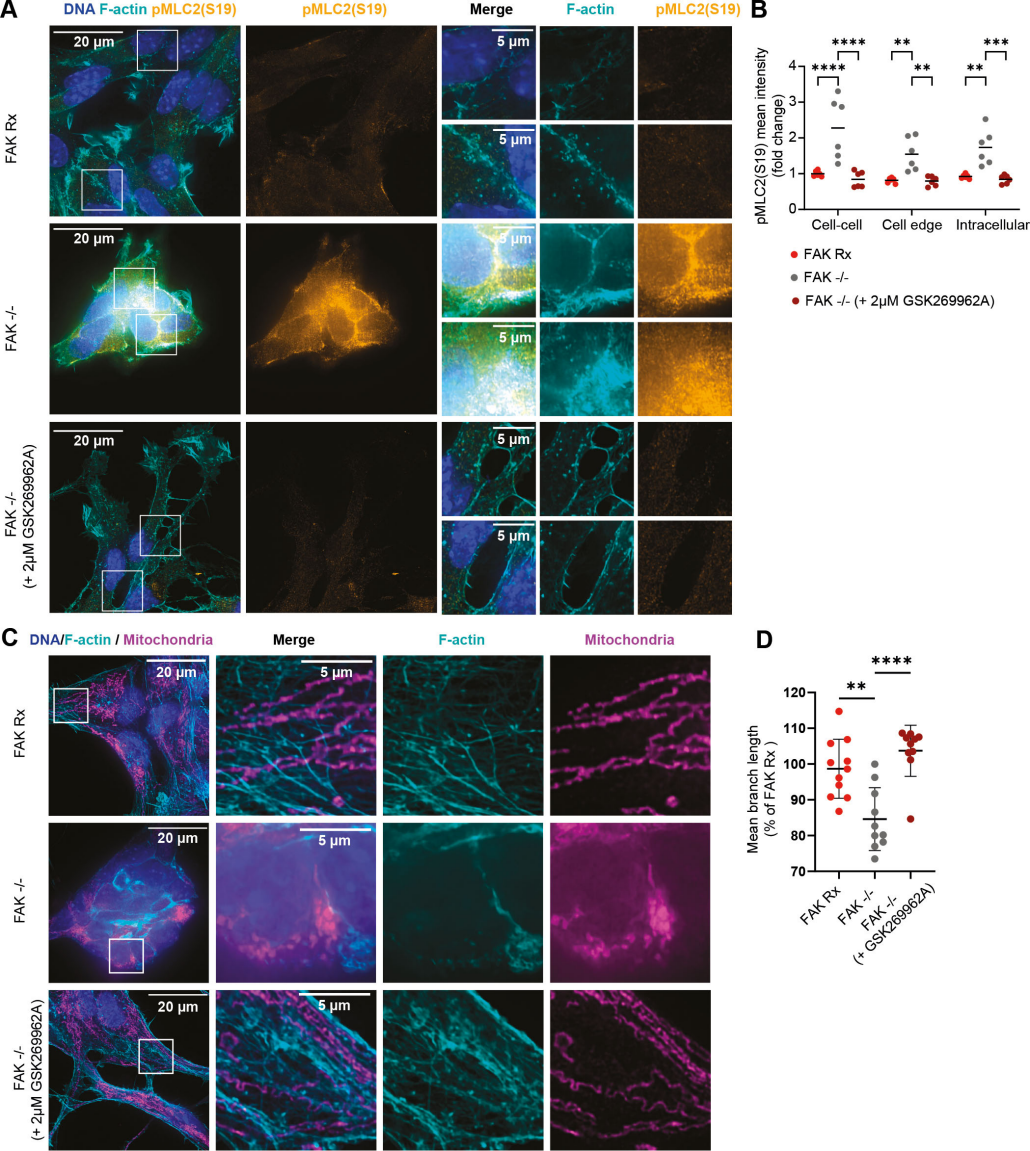
**FAK regulates mitochondrial morphology via the ROCK−pMLC2(S19) pathway.** (A) Representative super-resolution microscopy images of FAK Rx, FAK−/− cells treated with or without 2 µM GSK269962A ROCK inhibitor for 24 h, showing pMLC2(S19) (yellow) and the F-actin cytoskeleton (labelled with fluorophore-conjugated phalloidin, cyan); nuclei were stained with DAPI (blue). Boxed areas in main images are shown magnified in insets on the right, showing cell−cell contacts. Scale bars: 20 µm (full field of view), 5 µm (insets). (B) Quantification of pMLC2(S19) mean fluorescence intensity at cell−cell contacts, cell edge and intracellularly. *n*=2 independent experiments. Each dot represents one field of view. Error bars indicate the mean ±s.d. Statistics: two-way ANOVA followed by Tukey's multiple comparison test. **P<0.01, ***P<0.005, ****P<0.001. (C) Representative super-resolution microscopy images of FAK Rx, FAK−/− cells treated with or without 2 µM GSK269962A for 24 h, showing mitochondrial morphology (stained with MitoTracker Deep Red FM, magenta) and F-actin cytoskeleton (labelled with fluorophore-conjugated phalloidin, cyan); nuclei were stained with DAPI (blue). Boxed areas in main images are shown magnified in insets on the right. Scale bars: 20 µm (full field of view), 5 µm (insets). (D) Quantification of mitochondrial mean fragment (branch) length in FAK Rx and FAK−/− cells with or without 2 µM GSK269962A. *n*=2 independent experiments. Each dot represents one field of view. Error bars indicate the mean ±s.d. Statistics: one-way ANOVA followed by Tukey's multiple comparison test. **P<0.01, ****P<0.001.

Phosphorylation of the regulatory myosin light chain type II (MYL2, also known as MLC2) at serine residue (S19) [hereafter referred to as pMLC2(S19)], initiates actin-myosin interactions and assembles myosin filaments driving actomyosin contractility (Yu et al., 2016). We therefore used pMLC2(S19) as a surrogate measure of cellular actomyosin contractility. In order to localise pMLC2(S19) at a subcellular level in FAK-expressing and FAK-deficient cells, we used SoRa spinning disk microscopy and quantified pMLC2(S19) intensity at cell−cell contacts, the non-junctional cortex (cell edge), and the cytoplasm; ROIs were guided by the actin channel. We found that FAK−/− cells had a higher mean intensity of pMLC2(S19) at all of these subcellular locales, and noticed that pMLC2(S19) mean intensity was highest at cell−cell contacts with intense junctional F-actin (Fig. 4A,B). This implies that the increased cell−cell contacts formed as a result of FAK depletion are associated with highly localised F-actin and actomyosin contractility at the intercellular junctions. Next, we used inhibitors of ROCK, the kinase the maintains MLC2(S19) phosphorylation (Parsons et al., 2010). We treated FAK−/− cells with 2 μM of the ROCK inhibitor GSK269962A, which reduced pMLC2(S19) signals across the cells and induced cell spreading, suppressing cell−cell contacts and restoring a mesenchymal-like morphology (Fig. 4A,B). Taken together, these data demonstrate that pMLC2(S19) (and, by inference, actomyosin contractility) localizes to cell−cell contacts in FAK-deficient cells and that treating cells with a ROCK inhibitor enhances cell spreading and restores the mesenchymal-like morphology typical of FAK-expressing NPE cells.

To validate the antagonistic effect of FAK on ROCK signalling, we disrupted integrin-FAK signalling in FAK-expressing cells by comparing adherent (on laminin) and detached cells. We treated cells with the detachment agent Accutase for 30 min and maintained the cells in suspension within laminin-free medium and compared these cells to NPE cells grown on laminin when integrin-FAK signalling was active (Fig. S4A, lane 1). We then assessed FAK and ROCK activity by measuring pFAK(Y397) and pMLC2(S19) at appropriate time points by western blotting. We found a time-dependent decrease in pFAK(Y397) levels (normalised to total FAK) in suspended cells compared to cells spread on laminin (Fig. S4A,B). By contrast, pMLC2(S19) levels (normalised to total MLC2) were progressively increased in detached compared to spread cells over the time course of the experiment (Fig. S4A,B). This more subtle modulation of FAK signalling confirms the inverse relationship between integrin-FAK and ROCK signalling that we had observed upon FAK gene deletion.

Next, to address whether regulation of mitochondrial morphology through FAK is also dependent on its role in mechanotransduction, we used SoRa spinning disk microscopy to characterise mitochondrial morphology. We found that treating FAK-deficient cells with ROCK inhibitors GSK269962A (2 µM) or Y-27632 (20 µM) restored mitochondrial morphology to that exhibited by FAK-expressing cells, as judged by increased mitochondrial mean branch length (Fig. 4C,D and Fig. S4C,D). This implies that inhibiting ROCK-pMLC2(S19) signalling enhances mitochondria elongation, linking FAK-mediated actomyosin contractility to mitochondrial morphology.

### ROCK inhibitors increase glutamine oxidation and cell viability in FAK−/− cells

**Fig. 5.**
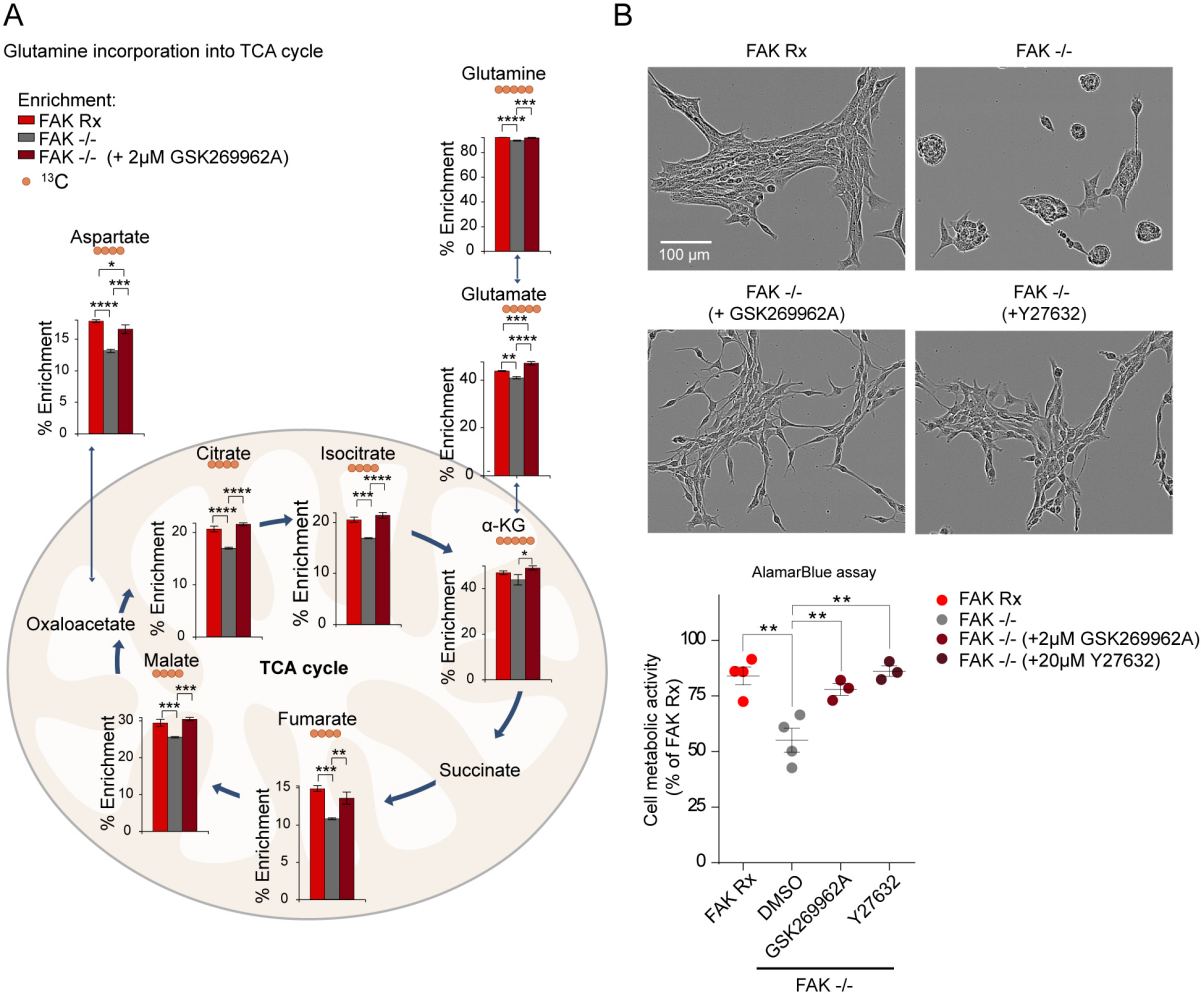
**Treating FAK−/− cells with ROCK inhibitor GSK269962A increases glutamine oxidation and cell metabolic activity.** (A) The atom fraction enrichment of glutamine-derived ^13^C in TCA intermediates in FAK Rx, FAK−/− and FAK−/− cells treated with or without 2 µM GSK269962A for 24 h followed by incubation for 3 h with ^13^C_5_ glutamine-supplemented medium. The main isotopologue of each metabolite is shown and plotted as the fraction of the sum of all isotopologues. α-KG, alpha-ketoglutarate. Error bars indicate the mean ±s.d. Statistics: one-way ANOVA with a Tukey multiple test correction (*n*=3 independent cultures on the same day). *P<0.05, **P<0.01, ***P<0.005, ****P<0.001. (B) Top: Representative phase-contrast images of FAK Rx, FAK−/− cells treated with and without ROCK inhibitors (+2 μM GSK269962A or +20 μM Y-27632) for 3 days. Bottom: Plotted is the metabolic activity of FAK Rx, FAK−/− cells treated with and without ROCK inhibitors (+2 μM GSK269962A or 20 μM Y-27632) for 3 days measured by alamarBlue assay. DMSO, vehicle control. Error bars indicate the mean ±s.e.m. Statistics: one-way ANOVA with a Dunnett multiple test correction (*n*≥3 independent experiments). **P<0.01.

We hypothesized that the observed restitution of mitochondrial morphology is associated with the restoration of normal glutamine oxidation found by re-expressing FAK in otherwise FAK-deficient cells. To test this, we analysed glutamine flux by using a ^13^C_5_-labelled glutamine tracer to analyse TCA cycle metabolites. We found that both ROCK inhibitors GSK269962A (2 µM) and Y-27632 (20 µM) increased glutamine oxidation to normal levels (Fig. 5A and Fig. S5A). Together, these data imply that FAK regulates mitochondrial morphology and glutamine oxidation by counteracting the ROCK-pMLC2(S19) signalling axis. We found that restoration of glutamine oxidation by ROCK inhibitor treatment rescued the loss of viability observed in FAK-deficient cells (Fig. 1D; Fig. 5B). These data indicate that FAK-mediated suppression of the ROCK/pMLC2(S19) pathway and actomyosin contractility not only control maintenance of the mesenchymal-like morphology and migration/invasion but are also inextricably linked to mitochondrial morphology, cell energy production and cell viability.

### FAK controls invasion, proliferation and survival *in vivo*

**Fig. 6.**
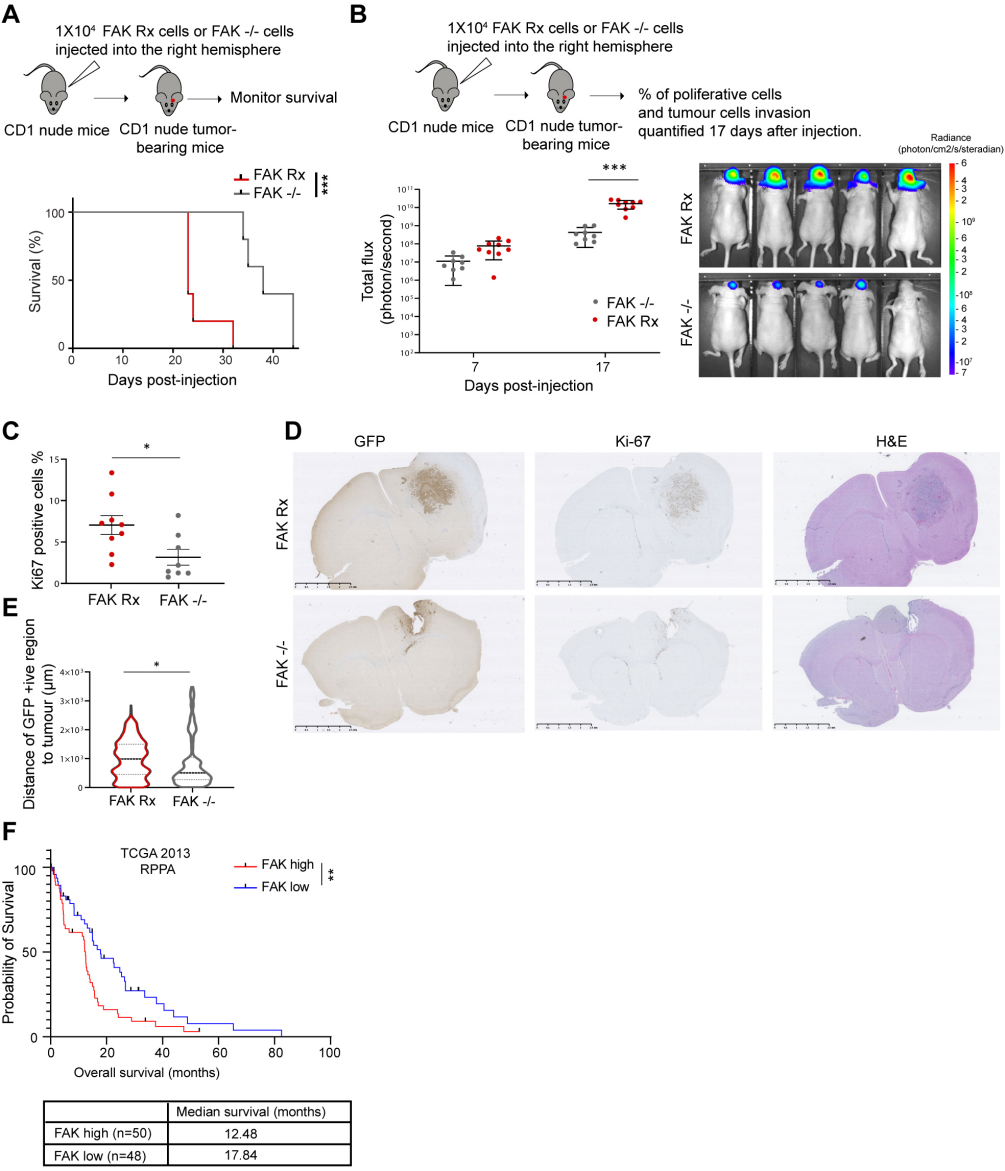
**FAK protein expression correlates with worse overall survival *in vivo* and in patients diagnosed with GBM.** (A) Kaplan–Meier curve of CD-1 nude mice bearing intracranial tumours from injecting FAK Rx or FAK−/− cells. Statistics: Mantel-Cox test, *n*=5 for each group. ***P<0.005. (B) Total body flux of CD-1 nude mice bearing intracranial tumours after injection of luciferase-expressing FAK Rx (*n*=9 mice) or FAK−/− cells (*n*=8 mice) at 7 and 17 days after injection measured by IVIS imaging. Error bars indicate the mean±s.e.m. Statistics: two-way ANOVA with Tukey post hoc adjustment for multiple comparisons. Right panel: representative *in vivo* bioluminescent images of CD-1 nude mice bearing tumours after injection of FAK Rx and FAK−/− cells at day 17. The heatmap superimposed over the mouse heads represents the radiance (photon/cm^2^/s/steradian). ***P<0.005. (C) Percentage of Ki-67+cells in sections of intracranial tumours after injection of FAK Rx (*n*=9 mice) or FAK−/− cells (*n*=8 mice) at 17 days after injection. Error bars indicate the mean ±s.e.m. Statistics: unpaired two-tailed *t*-test. *P<0.05. (D) Representative images of intracranial tumours stained for GFP or Ki-67, or H&E-stained, after injection of FAK Rx (*n*=9 mice) or FAK−/− cells (*n*=8 mice) at 17 days after injection. (E) Distance of GFP+ tumour cells to tumour edge in sections of intracranial tumours after injection of FAK Rx (*n*=4) or FAK−/− cells (*n*=4) at day 17 after injection. Mean and s.e.m. are shown. Statistics: unpaired two-tailed *t*-test. *P<0.05. (F) Kaplan–Meier curve showing the overall survival of patients diagnosed with GBM (*n*=98) stratified into two groups representing higher (*n*=50) and lower quartile (*n*=48) FAK protein levels measured by RPPA. Data obtained from the TCGA database were accessed using cBioPortal. Mean overall survival decreased in patients that had tumours with higher FAK protein levels. Statistics: log rank test. **P<0.01.

Loss of FAK led to a significant decline in key hallmarks of the malignant phenotype in the transformed neural stem cell model of GBM we used here, causing them to exhibit reduced invasion, migration, cell viability and suppressed glycolysis and glutamine oxidation rendering cells less energetic *in vitro*. To investigate whether this would translate to beneficial properties *in vivo*, we performed intracranial transplantation of GFP- and luciferase-expressing FAK−/− or FAK Rx cells into the right-side brain hemisphere of CD-1 nude mice. We found that mice injected with cells lacking FAK had significantly increased overall survival rates (Fig. 6A). CD-1 nude mice with intracranial tumours that had formed after injecting FAK Rx cells showed an increased total body flux of the luciferase reporter when compared to FAK−/− tumours at days 7 and 17 (Fig. 6B), suggesting that FAK expression increases tumour growth. This was associated with a higher percentage of Ki-67-expressing cells in sections of FAK-expressing intracranial tumours at 17 days (Fig. 6C,D). Moreover, FAK loss significantly reduced the distance NPE cells were able to invade healthy brain tissue from the tumour margin, a measure of their *in vivo* invasive capacity (Fig. 6E). This implies a role for FAK in cancer cells invasion *in vivo*.

Finally, to investigate the relevance of our findings to human GBM, we used data from the Cancer Genome Atlas (TCGA) to examine whether there is an association between FAK protein levels (measured by reverse-phase protein array) and survival in adult patients diagnosed with GBM. We identified a group of patients diagnosed with GBM with high (*n*=50) or low (*n*=48) FAK expression, representing those with higher or lower quartile FAK expression, respectively. Our analysis revealed an inverse correlation between FAK protein levels and overall patient survival (*P*=0.0088), consistent with FAK contributing to GBM pathogenesis in patients (Fig. 6F).

## DISCUSSION

Metabolic processes play critical roles in GBM as highlighted by the recently identified metabolic subtypes with associated metabolic vulnerabilities (Garofano et al., 2021; Zhang et al., 2024). Moreover, many GBM driver mutations, such as loss of the tumour suppressor *PTEN*, or amplification of receptor tyrosine kinase-encoding genes, such as *EGFR* or platelet-derived growth factor receptor A (*PDGFRA*), can enhance PI3K−AKT−mTOR signalling. This, in turn, confers metabolic plasticity, so GBM cells can tailor different metabolic pathways to their requirements (Huse and Holland, 2010). Aerobic glycolysis has been established as a metabolic pathway beneficial for tumour growth and, more recently, the TCA cycle has also been shown to be important for cancer cells both in mouse models and patients (Heiden et al., 2009; Ward and Thompson, 2012). The role of the ECM in modulating these pathways to enhance metabolic plasticity and resilience – including under therapeutic and mechanical stress – has begun to be elucidated, and is cell-type and tissue specific (Tharp et al., 2021; Papalazarou et al., 2020; Romani et al., 2022; Crosas-Molist et al., 2023). For example, mechanical cues originating in the ECM can influence ATP production and recycling, aiding the invasive migration of pancreatic cancer cells (Papalazarou et al., 2020). In addition, increased stiffness boosts glycolysis in human bronchial epithelial cells, while transformed non-small-cell lung cancer cells maintain high glycolytic rates despite changes in environmental mechanics (Park et al., 2020), thereby demonstrating cell- and tumour-context dependency. Furthermore, cell−ECM-mediated mechano-sensing and downstream signalling are also known to regulate mitochondrial structure and function, conferring resilience to redox stress in malignant human mammary epithelial cells (Tharp et al., 2021).

Despite these advances, it is still not known how specific adhesion proteins that function downstream of ECM−cell interactions influence cellular energetics and metabolic flexibility. The work we present here has uncovered a novel link by which a pivotal adhesion protein downstream of integrin engagement, namely FAK, is vital for optimal throughput of both glycolysis and the glutamine oxidation to ensure that cells from a transformed neural stem cells model of GBM have sufficient energy production. Deletion of the FAK gene or inhibition of FAK kinase activity in response to VS-4718, markedly impairs cellular energy output by suppressing both glycolysis and glutamine oxidation. This is important because glutamine is the major molecule that replenishes the TCA cycle in glioma cell lines and its uptake is highly enhanced, with minimal uptake in the surrounding brain tissue in both mice and humans diagnosed with GBM (Venneti et al., 2015). Our findings showed that FAK promotes glycolysis and glutamine oxidation, at least in part, by antagonizing ROCK-MLC2 signalling. This was supported by the observation that FAK depletion leads to increased phosphorylation of MLC2, including at cell–cell junctions, in cells that had been induced to undergo a mesenchymal-to-epithelial-like morphological transition. Furthermore, disruption of integrin–adhesion signalling by detaching FAK wild-type cells and culturing them in suspension revealed an inverse relationship between phosphorylation of FAK at Y397 and MLC2 activity measured by MLC2 phosphorylation at S19.

In addition to the suppression of cellular energetics via impaired glycolysis and glutamine oxidation in FAK-depleted transformed mouse neural stem cells, we found visible changes to mitochondrial morphology. Function of mitochondria is known to be closely connected to their structure, which can impact human health and disease in a context-dependent manner (Sprenger and Langer, 2019). We found that the role of FAK in promoting glutamine oxidation is associated with an elongated mitochondrial network. The changes in mitochondrial morphology in response to FAK depletion were dependent on FAK-mediated suppression of signalling to pMLC2(S19). Indeed, treating FAK-deficient cells with one of two distinct ROCK inhibitors (GSK269962A or Y-27632) restored mitochondrial morphology to the elongated form associated with increased glutamine incorporation into the TCA cycle. In keeping with this, RhoA activity has been shown to promote mitochondrial fission in cardiomyocytes by phosphorylating dynamin 1 like (DNM1L, also known as DRP1) at S616, leading to its re-localization to mitochondria (Brand et al., 2018); this is blocked by treatment with the ROCK inhibitor Y-27632 (Brand et al., 2018). Additionally, higher MLC2 activity has previously been associated with mitochondria fragmentation in human melanoma cells (Crosas-Molist et al., 2023). Taken together, we concluded that one of the key roles FAK has in the GBM stem cell model is to sustain cellular energetics via both glycolysis and glutamine oxidation, linked to mitochondrial structural changes that are accompanied by phosphorylation of MTFR1L on S235, which promotes mitochondrial dynamics (Tilokani et al., 2022). We propose this contributes to impaired tumour growth and overall survival *in vivo* when cells are implanted into the brains of recipient mice.

As mentioned, the work reported here used a mesenchymal-like GBM stem cell model; when converting these cells into more epithelial-like cells by deletion of the gene encoding FAK, both glycolysis and mitochondrial respiration are significantly impaired. However, previous studies using a pancreatic ductal adenocarcinoma (PDAC) epithelial cell line and an epithelial-like GBM cell line both cultured in serum-containing medium, rather than the serum-free stem cell medium we used here, suggests that FAK promotes glycolysis but suppresses OXPHOS under these conditions (Zhang et al., 2016; Che et al., 2021). When taken together with our work, these studies imply that the role of FAK in modulating the metabolism in epithelial-like cells differs from its role in mesenchymal-like cells. In this regard, we note that genome-wide CRISPR screens have shown that, in GBM stem cells, the gene encoding FAK is essential in the injury-response transcriptional state but not in the developmental transcriptional state (Richards et al., 2021; MacLeod et al., 2024). Injury response GBM stem cells map to the mesenchymal-like and glycolytic/plurimetabolic cell states, while developmental GBM stem cells, which are much less dependent on integrin signalling and FAK, map to non-mesenchymal-like cell states, such as ‘neural precursor’, ‘astrocytic’ and ‘oligodendrocyte progenitor’-like (Richards et al., 2021; MacLeod et al., 2024). Our data imply that mesenchymal-like cells are dependent on FAK for energy production.

Our findings offer one possible explanation for why genes that encode FAK and other integrin or their effector adhesion proteins – such as, for example, ITGB1 and ILK – are amongst the primary fitness genes identified in drop-out screens in the highly treatment-resistant mesenchymal-like subtypes/GBM cell states (Richards et al., 2021; MacLeod et al., 2024). The novel role we have identified for FAK in promoting metabolic pathways within mesenchymal-like cells implies that FAK controls the bioenergetic and biosynthetic requirements for the metabolic flexibility that characterises these GBM tumours. In turn, this likely contributes to tumour cell growth *in vivo* and their resistance to multiple therapeutics (Bhat et al., 2013). Our data imply that targeting FAK is likely to limit cellular energy production and metabolic flexibility of mesenchymal-like and glycolytic/plurimetabolic GBM tumours. Previous studies have suggested that inhibiting FAK can sensitize GBM cells to chemotherapy. Combining the FAK/Pyk2 inhibitor PF-56227 with temozolomide (TMZ) can enhance the effectiveness of TMZ in inhibiting tumour growth and spread, ultimately improving treatment outcomes (Ortiz-Rivera et al., 2023). Additionally, the use of the FAK autophosphorylation inhibitor Y15 alongside TMZ has been shown to synergistically reduce glioblastoma cell viability and to promote apoptosis (Golubovskaya et al., 2012). Based on the new role of FAK in modulating GBM stem cell energetics, we suggest that a combination of agents targeting FAK together with those that inhibit residual metabolic pathways in susceptible GBM subtypes may lead to enhanced therapeutic benefit.

## MATERIALS AND METHODS

### Experimental model and subject details

#### Cell culture

NPE cells were generated using CRISPR/Cas9 mutagenesis from neural stem cells of adult male C57BL/6-SCRM mice, as previously described (Gangoso et al., 2021). NPE cells were maintained in 5% CO_2_ at 37°C and were routinely tested for mycoplasma. Cell numbers were obtained using the Countess automated cell counter (Thermo Fisher Scientific) by Trypan Blue exclusion. NPE cells were cultured in Dulbecco's Modified Eagle’s Medium and Ham's Nutrient Mixture F12 (DMEM/F12; Sigma-Aldrich; #D8437) supplemented with 1.45 g/l D-Glucose (Sigma-Aldrich; #G8644), 120 μg/ml bovine serum albumin (BSA) fraction V solution (Gibco; #15260-037), 100 μM β-mercaptoethanol (Gibco; #31350-010), 1× Minimum Essential Medium non-essential amino acid (MEM-NEAA) solution (Gibco; #11140-035), 0.5× B-27 (Gibco; #17504-044) supplement, 0.5× N-2 supplement (Gibco; #15140-122), 10 ng/ml murine EGF (PeproTech; #315-09), 10 ng/ml human b-FGF (Pep Rotech; #100-18b), and 1 μg/ml laminin-I (R&D Systems; #3446-005-01). Cells were passaged every second day by dissociation with Accutase (Sigma-Aldrich; #A6964).

#### *In vivo* experiments

All treatments and procedures with mice were performed in accordance with protocols approved by Home Office UK guidelines in a designated facility under a project license PP7510272 held by V.G.B. at the University of Edinburgh. Up to six mice were caged in ventilated cages in a pathogen-free facility on a 12-h light/dark cycle with unlimited access to food and water. 6–15-week-old female CD-1 nude mice (Charles River, Strain number 086) were injected intracranially with 10,000 cells of either NPE-derived FAK−/− cells or NPE-derived FAK Rx cells suspended in 2 all neural stem cell medium following administration of isoflurane general anaesthesia. Mice were allocated to groups randomly. Mice were given analgesic buprenorphine subcutaneously during surgery and the analgesic carprofen was administered in drinking water for 48 h post surgery. Experimental mice were placed in a 25−30°C heat box during recovery from anaesthesia. Intracranial injections were performed in the mouse striatum at coordinates 0.6 mm anterior and 1.5 mm lateral to the bregma and 2.4 mm deep. Cells were prepared as previously described (Pollard et al., 2009). Bioluminescent imaging was performed while mice were under anaesthesia twice weekly by subcutaneous injection of 150 mg/kg luciferase using an IVIS Lumina S5 system (Revvity) for monitoring tumour growth. For survival experiments, mice were sacrificed by cervical dislocation upon the development of symptoms indicating the presence of a brain tumour, such as lethargy, instability, hunched posture and weight loss or until a maximum time point of 90 days post injection. For endpoint experiments, mice were sacrificed by cervical dislocation 17 days after cell implantation. Brains were collected immediately after sacrifice and fixed in 10% Neutral Buffered Formalin (Merck; HT501128-4L) overnight.

### Method details

#### Cell treatments

NPE cells were transfected by electroporation using a Lonza^®^ Nucleofector™ 2b device and the Lonza^®^ Mouse Neural Stem Cell Nucleofector™ Kit (Lonza; #VPG-1004) according to the manufacturer's instructions using the T-030 pulse code.

#### Plasmids

Plasmids for CRISPR/Cas9-mediated knockout of the gene encoding FAK were generated by cloning oligonucleotides encoding one single guide RNA (sgRNA) targeting the gene encoding FAK (Table S1) between the *Bbs*I restriction sites of the pSpCas9(BB)-2A-GFP (PX458) plasmid (Ran et al., 2013) (a gift from Feng Zhang; Addgene plasmid #48138; RRID: Addgene_48138). For re-expression of the gene encoding FAK, total RNA was extracted from NPE cells and reverse-transcribed to produce a cDNA library according to the manufacturer's protocols (Qiagen; #74104 and Thermo Fisher Scientific; #K1621). The gene encoding FAK was amplified from the cDNA library using specific primers containing a Kozak consensus sequence (Kozak, 1987) before the start codon with a 15 bp overhang (L_*Ptk2*_HD and R_*Ptk2*_HD; Table S1) to facilitate In-Fusion cloning (TakaraBio) into pQCXIN plasmid (a gift from Toby Hurd, Institute of Genetics and Cancer, The University of Edinburgh, UK) by In-Fusion cloning following the manufacturer's instructions.

#### CRISPR/Cas9 genome editing

1×10^6^ NPE cells were electroporated as described with 5 μg of pSpCas9(BB)-2A-GFP (PX458) with sgRNA targeting the gene encoding FAK (sgRNA: 5′-GCAGTAGTGAGCCAACCACCT-3′). This process was repeated twice at 48 h intervals. Cells were suspended in 5% BSA (Merck; #12659) in PBS and single cells were sorted using a BD FACSJazz™ cell sorter into wells of a 96-well cell culture plate. Cells were incubated in a humidified 37°C incubator at 5% CO_2_ until the formation of visible colonies. Colonies were passaged into two wells of a six-well plate, one of which was lysed directly in Laemmli buffer (50 mM Tris-HCl pH 6.8, 10% glycerol, 5% SDS, 5% β-mercaptoethanol, Bromophenol Blue), and processed for western blotting to screen for FAK depletion. Where FAK loss was detected, the remaining cells were passaged for further use.

#### DNA construct expression

For re-expression of the gene encoding FAK in NPE FAK−/− cells, 2×10^6^ cells were electroporated with 8 μg of FAK-pQCXIN. 48 h later, FAK-expressing cells were selected by addition of 0.5 mg/ml G418 (Merck; #A1720). G418 selection was continued for 2 weeks to select only cells that had stably integrated the FAK-pQCXIN construct. Expression of FAK in G418-selected cells was confirmed by western blotting. Selection was repeated every 3 months to ensure that the population retained FAK expression.

#### Cell lysis and western blotting

Cells were washed once with cold PBS and lysed in cold RIPA buffer (150 mM NaCl, 50 mM Tris-HCl pH 8, 1% v/v Triton X-100, 0.5% w/v sodium deoxycholate, 0.1% w/v sodium dodecyl sulphate) supplemented with cOmplete™ ULTRA protease inhibitor (Roche; #5892953001) and Phos STOP™ phosphatase inhibitor (Roche; #4906845001) cocktail tablets for 15 min at 4°C with gentle agitation. Crude extracts were collected into microcentrifuge tubes and centrifuged at 4°C for 15 min at 19,000 ***g***. Protein content was quantified using the Pierce™ BCA assay kit (Thermo Fisher Scientific; #23225) according to the manufacturer's protocol. 20 μg protein was diluted in Invitrogen novex NuPAGE LDS Sample Buffer (4×) and heated to 95°C for 7 min. Proteins were resolved by SDS-polyacrylamide gel electrophoresis using 4–15% Mini-PROTEAN^®^ TGX™ gels (BioRad; #4561086) and transferred to PVDF membranes (BioRad; #1704157) using the Trans-Blot Turbo semi-dry transfer system. Membranes were blocked by incubation with Tris-buffered saline (TBS) supplemented with 5% BSA and 0.1% TWEEN^®^ 20 (Millipore; #11332465001) (TBS-T) for 1 h at room temperature with gentle agitation. Primary antibodies were diluted in TBS-T supplemented with 5% BSA and incubated with membranes overnight at 4°C with gentle agitation. Membranes were washed three times with TBS-T at room temperature for 15 min with agitation. Secondary antibodies were diluted in TBS-T supplemented with 5% BSA and incubated with membranes for 45 min at room temperature with agitation. Membranes were washed a further three times in TBS-T and bound antibodies were visualised using the Clarity Western ECL substrate (BioRad; #1705061) with a ChemiDoc MP system (BioRad). Images were collected and quantified using the BioRad ImageLab software (v6.1).

#### *In vitro* 2D migration assay

500 cells/well were plated into an IncuCyte^®^ ImageLock 96-well plate (Sartorius; #BA-04856) and imaged using a 10× objective every 10 min for a period of 48 h. Single-cell tracking was performed using Fiji/ImageJ (v2.14.0/1.54f) (Schindelin et al., 2012) using Trackmate7 plugin (Ershov et al., 2022).

#### *In vitro* respirometry

7000 cells/well were seeded in a Seahorse XF24 cell culture microplate for 48 h (Part # 102342-100) after which medium was replaced with Seahorse XF medium (#103680-100) supplemented with 10 mM glucose (103577-100), 2 mM glutamine (103579-100), 1 mM pyruvate, 10 ng/ml murine EGF, and 10 ng/ml human b-FGF and incubated in a CO_2_-free incubator for 30 min before the assay. During respirometry, the following inhibitors were sequentially added via injection ports: oligomycin (1 μM final), FCCP (2 μM final) and antimycin A/rotenone (0.5 μM final). Concentrated stock solutions were prepared in Seahorse XF medium (#103680-100) for mitochondrial stress test compounds. XFe24 sensor cartridges containing 1 ml Seahorse XF Calibrant Solution/well were incubated for 16 h before the assay in a CO_2_-free incubator. Measurements were performed using Seahorse XFe24 Analyzer (Agilent) and data visualised and pre-processed by using the Wave software (v.2.6.3.5). Cells were dissociated with Accutase (Sigma-Aldrich; #A6964) and counted after the assay. Data were normalised to cell number. Oxygen consumption rate (OCR) traces were analyzed to derive mitochondrial parameters as follows. Non-mitochondrial oxygen consumption was defined as the minimum rate measured after rotenone/antimycin A injection. Basal respiration was calculated as the last rate measurment before oligomycin injection minus non-mitochondrial respiration. Maximal respiration was the maximum rate after FCCP injection minus non-mitochondrial respiration. Proton leak was the minimum rate after oligomycin injection minus non-mitochondrial respiration, and ATP-linked respiration was the last rate before oligomycin minus the minimum rate after oligomycin. Spare respiratory capacity was computed as maximal minus basal respiration, with spare capacity (%) equal to maximal/basal × 100. Coupling efficiency was calculated as (ATP-linked respiration rate / basal respiration rate) × 100.

#### Drug treatments

NPE cells were incubated with ROCK inhibitors as follows: 20 μM Y-27632 dihydrochloride (Tocris; #1254) or 2 μM GSK269962A (Cayman Chemical; #19180). Both compounds were prepared as DMSO stocks (Y-27632, 50 mM; GSK269962A, 10 mM), DMSO was matched to 0.04% (v/v) in all conditions; vehicle controls received 0.04% DMSO. Drugs were solubilised in DMSO to stock concentrations of 50 mM or 10 mM, respectively.

NPE cells were treated with either ROCK inhibitor for 24 h prior to assessing mitochondrial morphology (Fig. 4C and Fig. S4C,D) and glutamine oxidation (Fig. 5A and Fig. S5). For the alamarBlue assays, cells were treated with either ROCK inhibitor for 72 h (Fig. 5B).

For FAK inhibition, cells were treated with the FAK inhibitor VS-4718 at a final concentration of 300 nM or an equivalent volume of DMSO (final concentration 0.003%) for 48 h under standard culture conditions.

#### Liquid chromatography – mass spectrometry metabolomics

500,000 cells/well were seeded in a six-well plate and cultured for 48 h before metabolites were extracted with a solvent mixture (50% methanol, 30% acetonitrile, 20% H_2_O v/v) on dry ice for 15 min with agitation. The extraction solution was then centrifuged at 16,100 ***g*** for 10 min at 4°C. The supernatants were stored at −75°C prior to liquid chromatography–mass spectrometry (LC−MS) analysis. Plates were left in a fume hood to dry and protein quantification was performed by using the Lowry assay.

Samples were randomised and metabolites were analysed on a Dionex Ultimate 3000 UHPLC (Thermo Fisher Scientific) coupled to a Q Exactive Hybrid Orbitrap MS (Thermo Fisher Scientific). Hydrophilic interaction liquid chromatography (HILIC) using a ZIC-pHILIC analytical column (2.1×150 mm, SeQuant-MerckMillipore) coupled with a guard column (2.1×20 mm) was used for chromatographic separation of metabolites. A gradient using mobile phases A [20 mM ammonium carbonate, 0.01% (v/v) ammonium hydroxide] and B (acetonitrile) was used. The gradient was set from 95% to 5% B over 20 min and re-equilibrated to initial conditions for 7 min. The flow rate was 200 ml/min, and the temperature was at 45°C. The injection volume was 5 µl. MS data were acquired in positive/negative polarity switch mode in the m/z range of 70–900 Da, with a resolving power of 70,000 full width at half maximum (FWHM). Metabolites were identified by matching accurate mass measurements (<5 ppm) and retention times to an in-house library of standards. Skyline 21.2.0.568 (Pino et al., 2020) was used to quantify the metabolites by integrating the area under the curve and normalizing the values to total protein content. Metaboanalyst 5.0 (Xia et al., 2009) was used to perform log transformation (base 10), auto scaling (data mean-centred and divided by the standard deviation of each variable) and KEGG metabolic pathway enrichment analysis.

#### ^13^C_6_-glucose and ^13^C_5_-glutamine LC-MS metabolomics

500,000 cells/well were seeded in a six-well plate and cultured for 48 h before medium was exchanged with medium containing ^13^C_6_-Glucose (Sigma-Aldrich; #389374) at 4.5 g/l and incubated at 37°C for 1 h for ^13^C_6_-glucose tracing. For glutamine tracing medium was replaced with medium containing ^13^C_5_-Glutamine (Sigma-Aldrich; **#**605166) at 0.365 g/l and incubated at 37°C for 3 h. Cells were washed twice with PBS and metabolites were extracted with a solvent mixture [50% methanol, 30% acetonitrile and 20% H_2_O (v/v)] on dry ice for 15 min with agitation. The extraction solution was then centrifuged at 16,100 ***g*** for 10 min at 4°C. The supernatants were stored at −75°C prior to LC-MS analysis. Metabolites were analysed as described above. Isotope natural abundance was corrected and percent enrichment of isotopologues was calculated using AccuCor package in R.

#### Manipulation of integrin-FAK signalling

NPE cells were seeded onto either laminin-coated surfaces (1 µg/ml) or 0.075% poly(2-hydroxyethyl methacrylate) (poly-HEMA) surfaces, which do not support integrin signalling, and cultured for 24 h. ^13^C_5_-glutamine isotope tracing was performed as described above under ‘^13^C_6_-glucose and ^13^C_5_-glutamine LC-MS metabolomics’.

NPE cells were seeded on laminin-coated surfaces (1 µg/ml) and cultured for 24 h. To disrupt integrin−FAK signalling, cells were detached using Accutase for 30 min. Following detachment, cells were centrifuged (300 ***g***) and resuspended in laminin-free medium. At specified time points (0 min, 30 min, 60 min, 90 min and 120 min after Accutase exposure), cells were harvested, lysed in RIPA buffer and protein concentrations were determined. Levels of phosphorylated FAK (pFAK Y397), phosphorylated MLC2 [pMLC2(S19)], total FAK and total MLC2 were analysed by western blotting.

#### Proteomics

Cells were lysed with the protein aggregation capture (PAC) lysis buffer [5% SDS, 100 mM Tris pH 8.5, 1 mg/ml chloroacetamide, 1.5 mg/ml tris(2-carboxyethyl) phosphine (TCEP)], heated at 95°C for 30 min followed by sonication. Lysates were then randomised and added to a KingFisher 96-well deep-well plate and prepared for an 8 h digest protocol on the KingFisher Duo (Thermo Fisher Scientific). Lysates were added to Row G with MagReSyn HILIC beads (ReSyn Biosciences) and acetonitrile was added to a final concentration of 70%. Rows D, E and F were filled with 95% acetonitrile and rows B and C were filled with 70% ethanol. The digest buffer (1 µg/ml MS grade trypsin in 50 mM triethylammonium bicarbonate) was added to Row A. Desalted peptides were then loaded onto 25 cm Aurora Columns (IonOptiks, Australia) using a RSLC nano uHPLC system connected to a Fusion Lumos mass spectrometer (Thermo Fisher Scientific). Peptides were separated by a 70 min linear gradient from 5% to 30% acetonitrile, 0.5% acetic acid. The mass spectrometer was operated in DIA mode, acquiring a MS 350−1650 Da at 120 k resolution followed by MS/MS on 45 windows with 0.5 Da overlap (200–2000 Da) at 30 k with a NCE setting of 28. The output was analysed on DIA-NN (v.1.8.2 beta 27) with the *Mus musculus* FASTA. The precursor m/z range was set to 350–1650 and the fragment ion range was set to 200−2000. Label-free quantification (LFQ) intensities for proteins quantified in at least three biological replicates in at least one experimental group were binary-logarithm transformed. Missing values were inputted from a width-compressed, down-shifted Gaussian distribution by using Perseus (version 1.6.15.0) (Tyanova et al., 2016).

#### Phosphoproteomics

Cells were lysed and proteins were digested as described above. Zr-IMAC beads were then used for phosphopeptide enrichment. The beads were suspended in a binding buffer [0.1 M glycolic acid, 85% acetonitrile and 5% trifluoroacetic acid (TFA)] and added to row G of the KingFisher deep-well plates. Another wash with the binding buffer was conducted in row F for equilibration of the beads. The digested peptides were added to binding buffer for a final volume of 200 µl. Two subsequent washes were conducted with 80% acetonitrile, 5% TFA and 0.1 M glycolic acid. A final wash was conducted with 10% acetonitrile, 0.2% TFA and 0.1 M glycolic acid. Phosphopeptides were eluted in 1% NH_4_OH, loaded on Evotips and analysed using a timsTOF single cell proteomics (SCP) (Bruker) coupled with an Evosep LC system on the Whisper 40 SPD method (Evosep Biosystems).

#### Immunofluorescence microscopy

Coverslips were washed overnight in 1 M hydrochloric acid at 65°C before being rinsed twice with deionised H_2_O and stored in 70% ethanol. Prior to plating cells, coverslips were washed in PBS and coated with 1 μg/ml laminin-I (R&D Systems; #3446-005-01) at 37°C for 1 h. 5×10^4^ cells were plated on coated coverslips and allowed to adhere for 48 h. Cells were fixed by replacing the medium with fixing buffer (3.7% formaldehyde/100 mM PIPES/10 mM EGTA/1 mM MgCl_2_/0.2% v/v Triton X-100) and incubation at 37°C for 15 min. Cells were washed once with TBS and incubated in 0.1 M glycine (Sigma-Aldrich; #G8898) in TBS for 10 min at room temperature to quench excess formaldehyde before an additional wash with TBS. Permeabilisation was performed with TBS-T for 5 min at room temperature. Cells were washed once in TBS supplemented with 0.05% Triton X-100 (v/v) and then blocked with TBS supplemented with 2% BSA and 0.1% Triton X-100 (v/v) for 1 h at room temperature. Primary antibodies were diluted in blocking buffer and incubated with cells overnight at 4°C, followed by three washes with TBS-T for 5 min each at room temperature with gentle agitation. Secondary antibodies and Alexa Fluor 488 phalloidin (Thermo Fisher Scientific; #A12379, working concentration 165 nM) were diluted in blocking buffer and incubated with cells in the dark for 45 min at room temperature. Cells were washed a further three times in the dark in TBST at room temperature for 5 min each with gentle agitation, followed by a final wash with deionised H_2_O. Cells were mounted with ProLong™ Glass Antifade Mountant with NucBlue™ (Invitrogen; #P36981) for nuclear staining. Cells were imaged on a Nikon CSU-W1 SoRa spinning disk microscope using 405 nm, 488 nm, 568 nm and 647 nm lasers with 100× oil immersion objective. Images were acquired using NIS-Elements AR 5.30.03 (Build 1549) or NIS-Elements AR 6.10.01 (Build 2027) software. To acquire *Z*-stacks, 4 µm thickness from the cell bottom were imaged with 0.1 µm or 0.2 µm step size. Images were denoised and deconvolved using NIS-Elements AR 5.21.03 (Build 1489) or NIS-Elements AR 6.10.01 (Build 2027) software and processed using Fiji/ImageJ (v2.14.0/1.54f) (Schindelin et al., 2012). ‘Enhance Contrast’ was used to help visualize images, except for Fig. 4A/pMLC2(S19) channel, where the minimum and maximum displayed values were kept the same for all conditions compared.

#### MitoTracker staining

MitoTracker Deep Red FM (Invitrogen; #M22426) was solubilized in DMSO at a stock concentration of 100 μM and stored at −20°C in aliquots. MitoTracker stock solution was diluted in medium to a concentration of 400 nM, which was added directly to cell culture medium already in the culture, yielding a final concentration of 200 nM. After 45 min incubation cells were fixed and permeabilised as described above. To extract mitochondria network features the *Z*-plane closest to the substratum, with well-resolved mitochondria, was chosen. Enhance Contrast with 0.1% saturation was used for all conditions and mitochondria thresholds were generated using Mitochondria analyser (v2.3.1) (Chaudhry et al., 2020) in Fiji/ImageJ (v2.14.0/1.54f) (Schindelin et al., 2012) through weighted mean method, 1.25 µm block size and C-value=9. Morphological mitochondria analysis was performed on the binarized images by using the same plugin on a field-of-view basis.

#### Immunohistochemistry

Brain samples from mice were fixed in 10% Neutral Buffered Formalin for 16 h and then transferred to 70% ethanol. Brain samples were then cut into 4 coronal sections before embedding in paraffin blocks. For each brain sample, 8-μm sections were prepared for hematoxilin and eosin (H&E) staining. For immunohistochemistry staining, 8-μm sections were mounted on glass slides. Paraffin removal from sections was achieved by washing samples twice in xylene for 5 min. Sections were rehydrated by incubation in decreasing ethanol solutions (100%, 75% and 50%) for 3 min each followed by rinsing with H_2_O. Tissue sections were boiled for 5 min in 10 mM sodium citrate buffer (0.825 M sodium citrate, 0.175 M citric acid pH 6.0) for antigen epitope retrieval. Sections were then rinsed with H_2_O and washed in TBS-T twice for 5 min each. Sections were incubated with Dako REAL peroxidase block solution (Agilent; #S2023) for 5 min and rinsed with H_2_O. For protein block, sections were treated with serum-free protein block solution (Agilent; #X0909) for 10 min. Primary antibodies were prepared in antibody diluent (Agilent; #S3022) and incubated with sections overnight at 4°C. Sections were washed three times in TBS-T for 5 min each and incubated with DAKO EnVision-HRP rabbit/mouse (Agilent; #K5007) for 45 min at room temperature. Sections were washed three times in TBS-T for 5 min each and developed with DAB-chromogen (Agilent; #K3468) at 1:50 dilution for 10 min followed by rinsing with H_2_O. Sections were then immersed in Mayer's Hematoxylin solution (Agilent; #S3309) for 2 min at room temperature and rinsed with H_2_O before incubation with Scott’s Tap Water Substitute (3.5 g/l sodium bicarbonate, 20 g/l magnesium sulphate) for 2 min and a final rinse with H_2_O. Sections were dehydrated by incubation in ethanol solutions (50%, 75% and 100% ethanol) for 3 min and washed in xylene twice for 5 min. Slides were mounted with DPX mountant (Sigma-Aldrich; #06522). Images of stained sections were obtained using a Nanozoomer slide scanner (Hamamatsu Photonics). Ki-67 and GFP stainings were analysed using QuPath software (version 4.0.3). GFP staining was used to distinguish tumour regions.

#### Antibodies

Antibodies used for western blotting were anti-FAK (Cell Signaling Technology; #3285, 1:1000), anti-Phospho-FAK (Tyr397) (Cell Signaling Technology; #3283, 1:1000), anti-NF1 (Bethyl; #A300-140A, 1:2000), anti-PTEN (Cell Signaling Technology; #9559, 1:1000), anti-EGFR (Cell Signaling Technology; #4267, 1:1000), anti-COX IV (Cell Signaling Technology; #4850, 1:1000), anti-cofilin (Cell Signaling Technology; #5175, 1:1000), anti-Glut1 (Cell Signaling Technology; #73015, 1:1000), anti-Enolase 2 (Cell Signaling Technology; #24330, 1:1000). Primary antibodies used for immunofluorescence microscopy were mouse anti-α-Tubulin (Cell Signaling Technology; #3873, 1:1000), and mouse anti-phospho-Myosin Light Chain 2 (Ser19) (Cell Signaling Technology; #3675, 1:200). Secondary antibodies used for immunofluorescence were Alexa Fluor™ 568-conjugated anti-mouse IgG (Invitrogen; #A-11004, all 1:400). Antibodies used for immunohistochemistry were anti-GFP (ChromoTek; #PABG1-20; 1:100) and anti-Ki-67 (Cell Signaling Technology; #12202, 1:1000).

#### Overall survival of patients diagnosed with GBM

Survival data of patients diagnosed with GBM were correlated with FAK protein expression (RPPA data) using data from the GBM TCGA database (Brennan et al., 2013) which was accessed through cBioPortal (de Bruijn et al., 2023; Gao et al., 2013; Cerami et al., 2012). Patients diagnosed with GBM were divided into two groups according to FAK protein levels. FAK-high and FAK-low groups representing patients with upper (*n*=50) and lower quartile (*n*=48) FAK protein expression levels, respectively.

#### Computational methods

Where R packages were used for analysis, this was performed using R (v4.4.1). Data were plotted using GraphPad Prism 9 (v9.4.0), R packages; gplots (v3.2.0) (Warnes et al. 2024), ggplot2 [ggplot2: Elegant Graphics for Data Analysis (3e)] and RColorBrewer. ^13^C_6_-glucose and ^13^C_5_-glutamine isotope tracing data were plotted using Escher-Trace (Kumar et al., 2020).

#### Quantification and statistical analysis

No statistical methods were used to predetermine the sample size. The investigators were not blinded to allocation during experiments and outcome assessment. Figure legends contain biological and technical replicate information. Plots include each data point; error bars indicate the mean +/±s.d. or the mean +/±s.e.m. Statistical analyses were performed in GraphPad Prism 9 (v9.4.0) or R (v4.4.1) or Metaboanalyst 5.0 (Xia et al., 2009). GSEA analysis was performed using the GSEA software (v4.3.1) (Subramanian et al., 2005). *P*-values are represented as *P<0.05, **P<0.01, ***P<0.005 or ****P<0.001 between the indicated groups.

## Supplementary Material

10.1242/dmm.052634_sup1 Supplementary information
