# Supplementary material for: FAK modulates glioblastoma stem cell energetics via regulation of glycolysis and glutamine oxidation
Source: Dis Model Mech. 2025 Nov 28;18(11):dmm052634. doi: 10.1242/dmm.052634 (PMC12690527; doi:10.1242/dmm.052634)
Supplement: Supplementary information [file dmm-18-052634-s1.pdf]

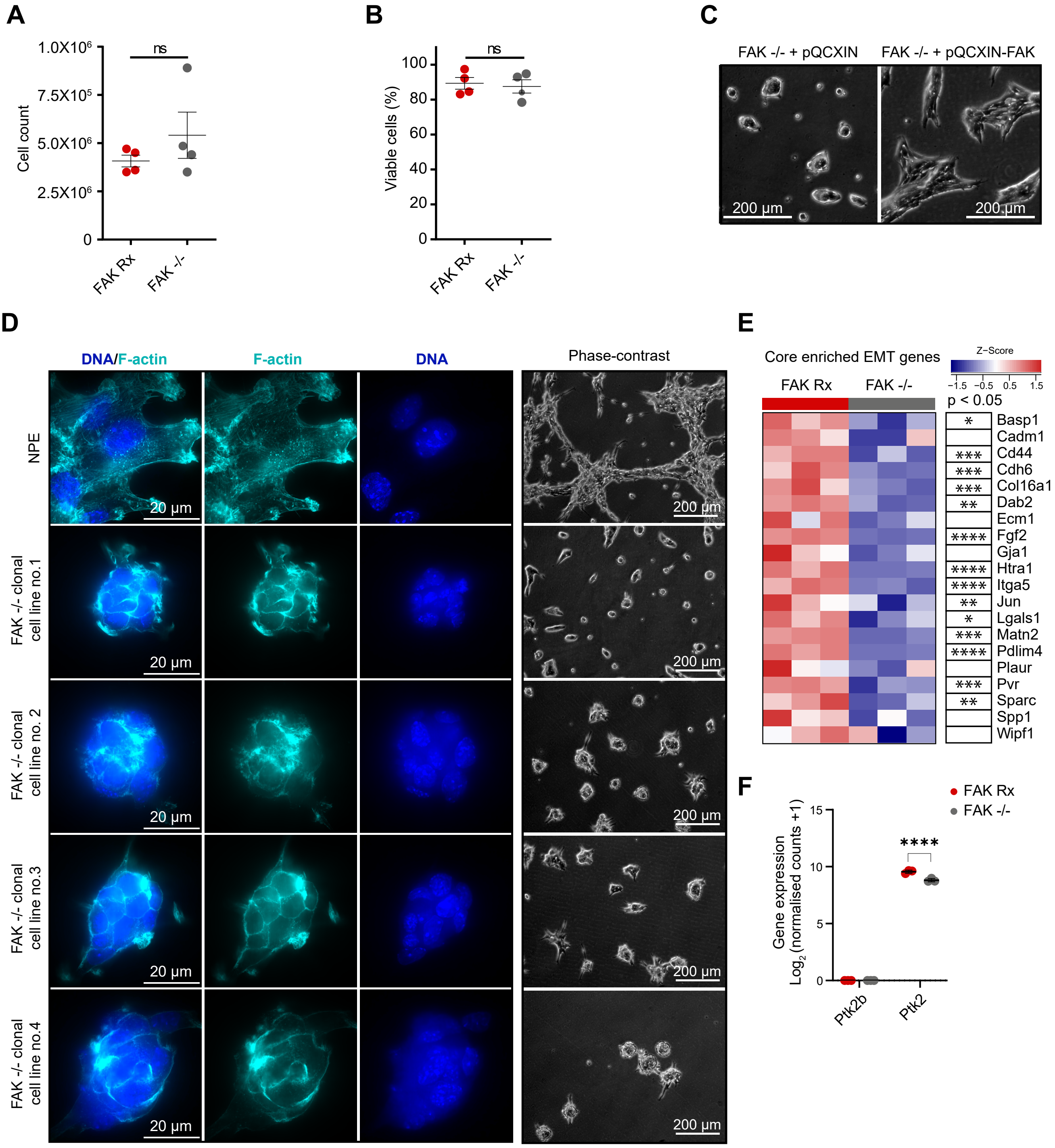

Fig. S1. Genetic deletion of FAK drives phenotypic and transcriptional changes in a mouse model of GBM stem cells.(A) Cell count of FAK Rx and FAK -/- cells cultured for 3 days under normal culture conditions. Mean and SEM are shown. Statistics: unpaired two-tailed t-test (n=4). (B) Cell viability by trypan blue exclusion of FAK Rx and FAK -/- cells cultured for 3 days under normal culture conditions. Mean and SEM are shown. Statistics: unpaired two-tailed t-test (n=4).

(C) Representative phase-contrast images showing cell morphology of FAK -/- cells 48h after nucleofection with either empty pQCXIN vector or pQCXIN-FAK vector, prior to selection for G418 resistance. The scale bar is 200 µm.

(D) Left panel: representative super-resolution microscopy images for NPE and NPE FAK -/- clonal cell lines showing the F-actin cytoskeleton labelled with fluorophore-conjugated Phalloidin (green), and nuclei labelled with 40,6-diamidino-2-phenylindole (DAPI; blue). Contrast was uniformly enhanced across all images to improve visualization. The scale bar is 20 µm. Right panel: representative phase-contrast images for NPE and NPE FAK -/- clonal cell lines. The scale bar is 200 µm.

(E) Heatmap of label-free quanification (LFQ) mass spectrometrey intensity of proteins corresponding to EMT core enriched genes in FAK Rx cells and FAK -/- cells. Each square represents an individual replicate (n = 3 independent cultures on the same day). Statistics: unpaired two-tailed t-test.

(F) Logarithm transformation (Log) of Ptk2b and Ptk2 (normalised counts+1) in FAK Rx and FAK -/- cells. Mean and SD are shown. Normalized counts were obtained using DESeq2 (negative binomial model, Wald test), with p-values adjusted for multiple testing (Benjamini-Hochberg method).

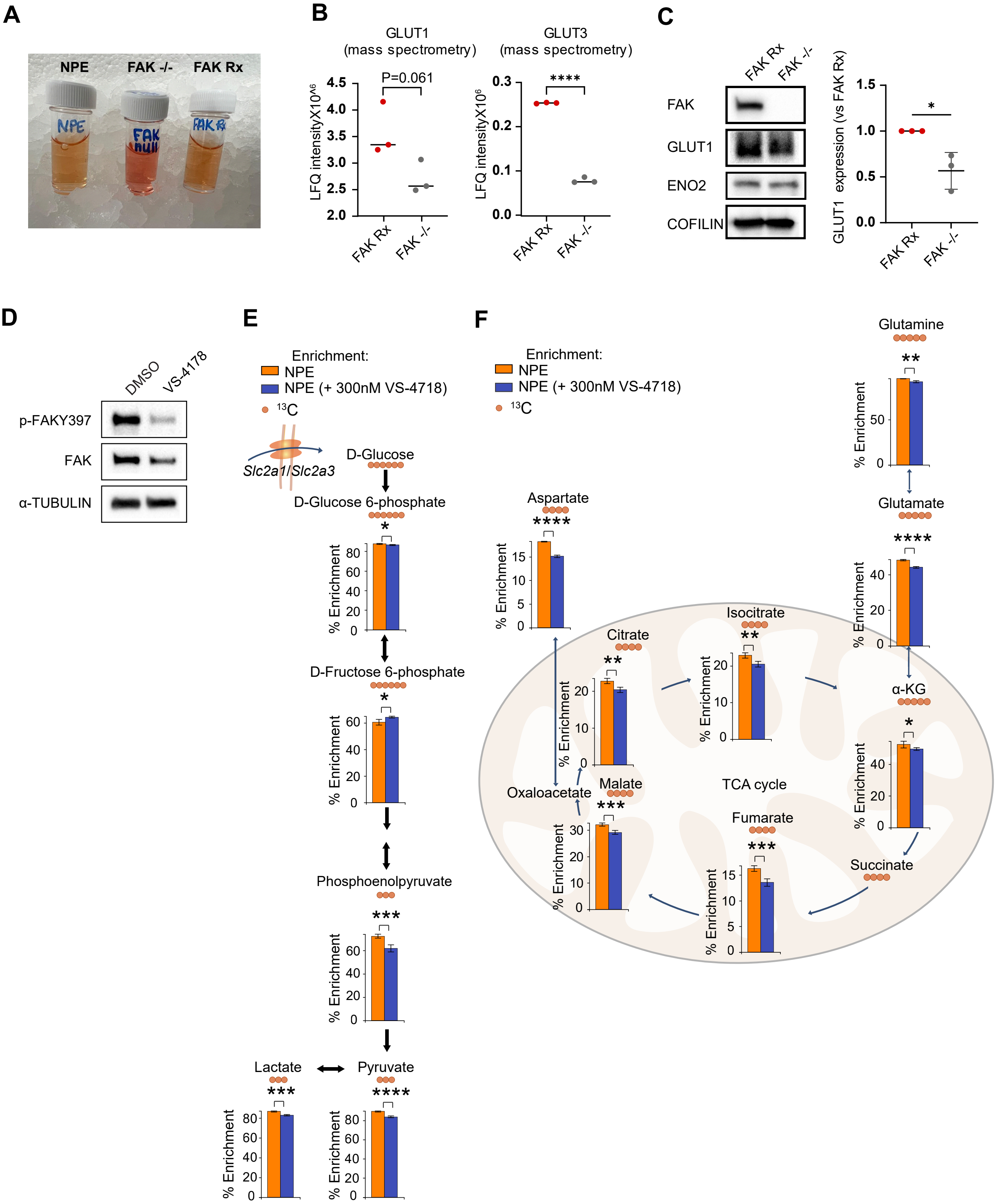

Fig. S2. FAK loss is associated with less acidic culture media and reduced Glut1 and Glut3 expression.
(A) Representative images of NPE, FAK -/- and FAK Rx culture media, containing the pH indicator phenol red.
(B) Label-free quanification (LFQ) mass spectrometrey intensity of GLUT1 and GLUT3 in FAK Rx and FAK -/- cells. Statistics: unpaired two-tailed t-test.
(C) Western blot showing FAK, GLUT1, ENO2 expression. COFILIN was used as a loading control. Right panel; quantification of normalised GLUT1 expression from 3 independent experiments. Statistics: unpaired two-tailed t-test.
(D) Western blot analysis showing expression of p-FAK Y397 and FAK in NPE cells treated with and without FAK inhibitor (+300nM VS-4718) for 48h. COFILIN was used as a loading control.
The atom fraction enrichment of glucose-derived ^{13}C in glycolysis intermediates (E) and glutamine-derived ^{13}C in TCA cycle intermediates (F) in NPE cells treated with and without FAK inhibitor (+300nM VS-4718) for 48h. The cells were incubated with $^{13}C_6$ glucose for 1h (E) or with $^{13}C_5$ glutamine for 3h (F). The main isotopologue of each metabolite is shown and plotted as the fraction of the sum of all isotopologues. Mean and SD are shown. Statistics: unpaired two-tailed t-test (n = 4 independent cultures on the same day).

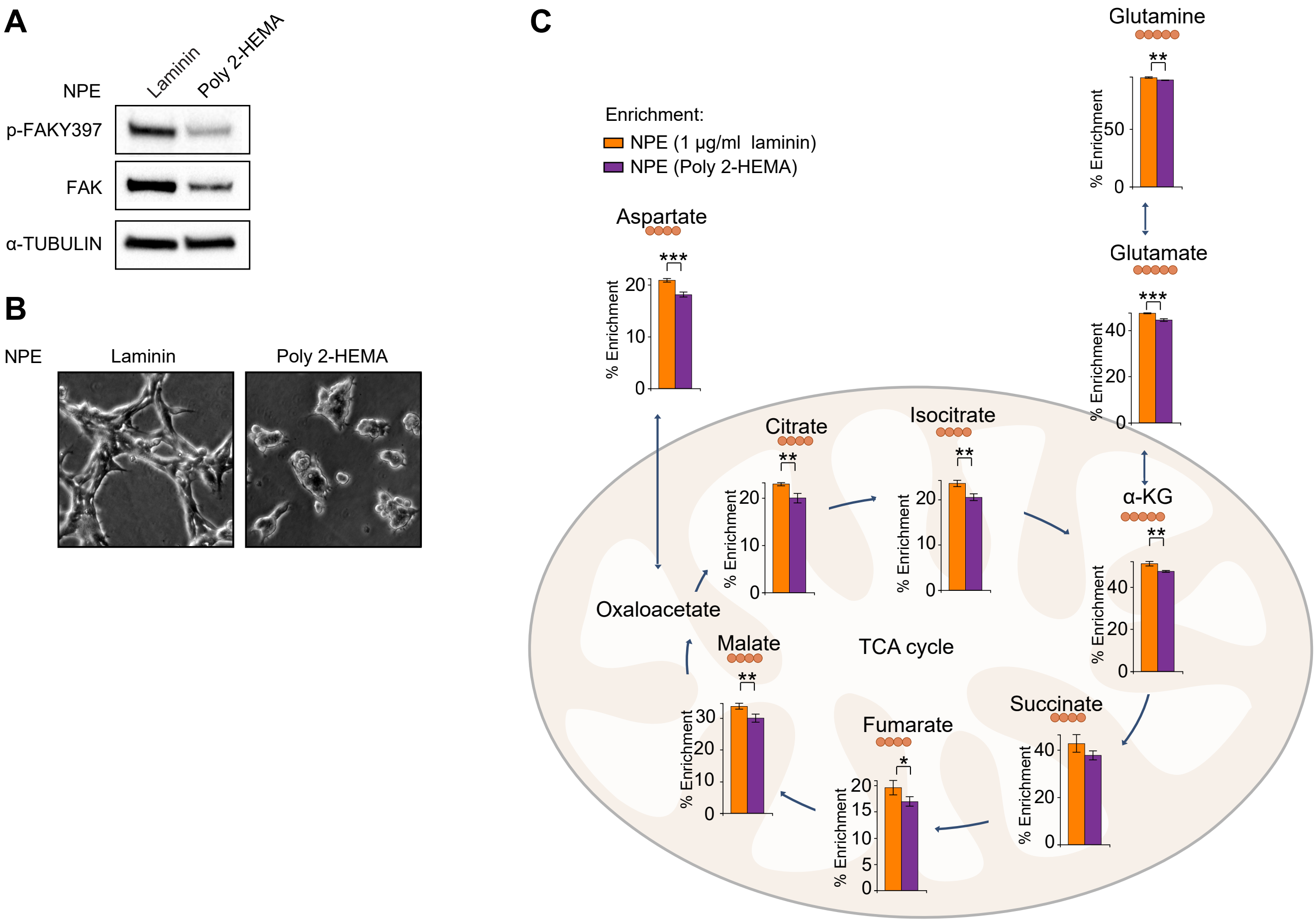

Fig. S3. Plating NPE cells on adhesion antagonist Poly 2-HEMA suppresses FAK and glutamine oxidation.

(A) Western blot analysis showing expression of p-FAK Y397 and FAK in NPE cells plated on 1 µg/ml laminin and %0.075 Poly 2-HEMA for 48h. α-TUBULIN was used as a loading control.

(B) Representative phase-contrast images of NPE cells plated on 1 µg/ml laminin and %0.075 Poly 2-HEMA for 48h.

(C) The atom fraction enrichment of glutamine-derived ^{13}C in TCA cycle intermediates in NPE cells plated on 1 µg/ml laminin and %0.075 Poly 2-HEMA for 48h. The cells were incubated with $^{13}C_5$ glutamine for 3h (C). The main isotopologue of each metabolite is shown and plotted as the fraction of the sum of all isotopologues. Mean and SD are shown. Statistics: unpaired two-tailed t-test (n = 4 independent cultures on the same day).

Disease Models & Mechanisms • Supplementary information

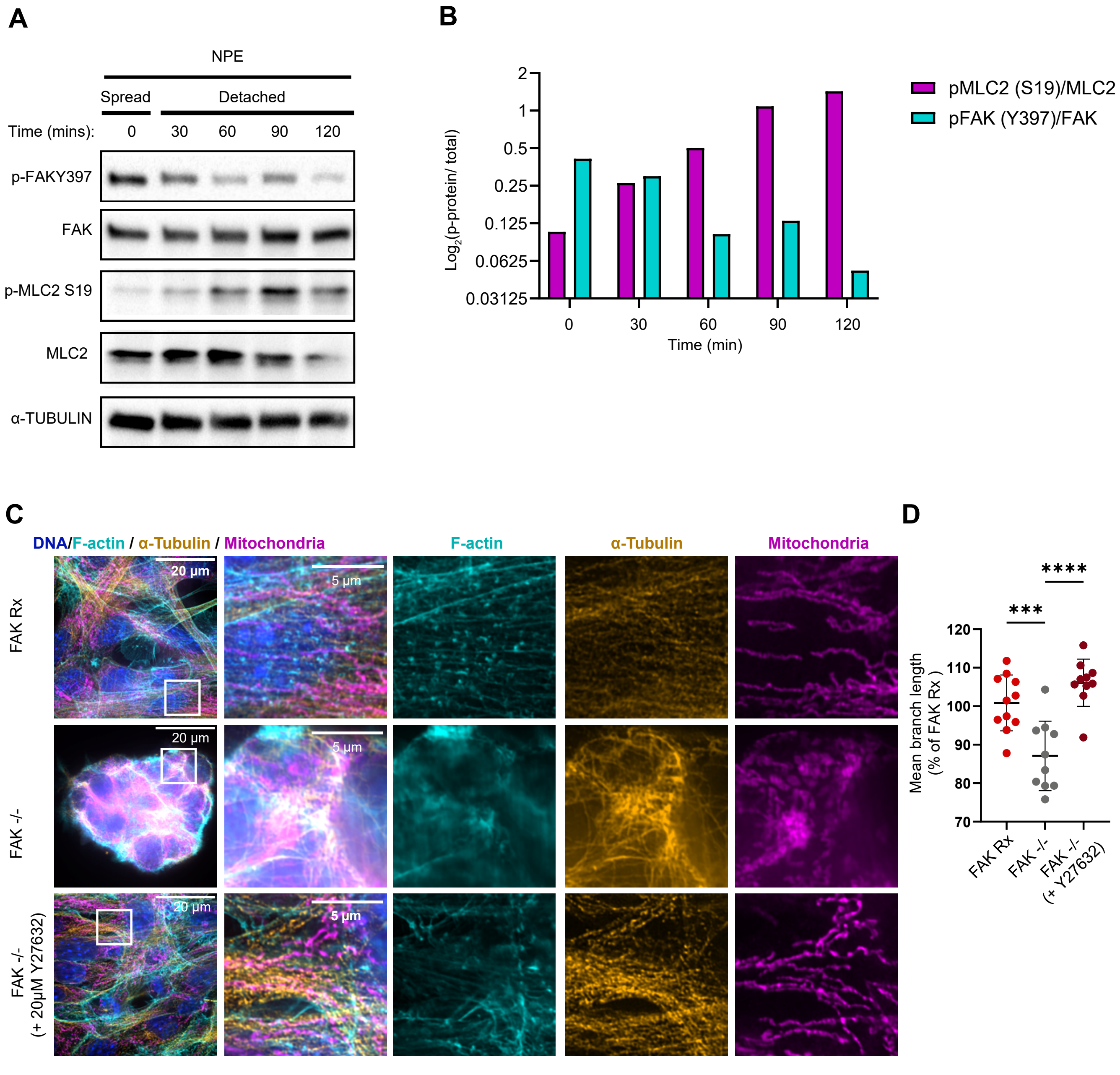

Fig. S4. FAK regulates mitochondria morphology through ROCK-pMLC2 S(19) pathway.
(A) Representative western blot showing levels of p-FAK (Y397), total FAK, p-MLC2 (S19), and total MLC2 in spread NPE cells plated on laminin (1 µg/ml), and in detached NPE cells collected at different time points after Accutase treatment (30 min) followed by suspension culture in laminin-free medium. Time indicates the period elapsed since Accutase exposure. α-TUBULIN was used as a loading control.
(B) Bar plot quantifying normalized p-MLC2 (S19) (magenta) and p-FAK (Y397) (cyan) levels corresponding to the samples shown in (A). Each bar represents an individual culture.
(C) Representative super-resolution microscopy images for FAK Rx, FAK -/- cells treated with or without 20 µM Y27632 for 24 h, showing mitochondrial morphology stained with MitoTracker Deep Red FM (magenta), the F-actin cytoskeleton labelled with fluorophore-conjugated phalloidin (cyan), α-Tubulin (orange) and nuclei labelled with DAPI (blue). Scale bar is 20 µm in the full field of view and 5 µm in insets.
(D) Quantification of mitochondrial mean fragment length in FAK Rx and FAK -/- cells with or without 20 µM Y27632. n = 2 independent experiments. Each dot represents a field of view. Mean and SD are shown. Statistics: one way ANOVA followed by Tukey's multiple comparisons test.

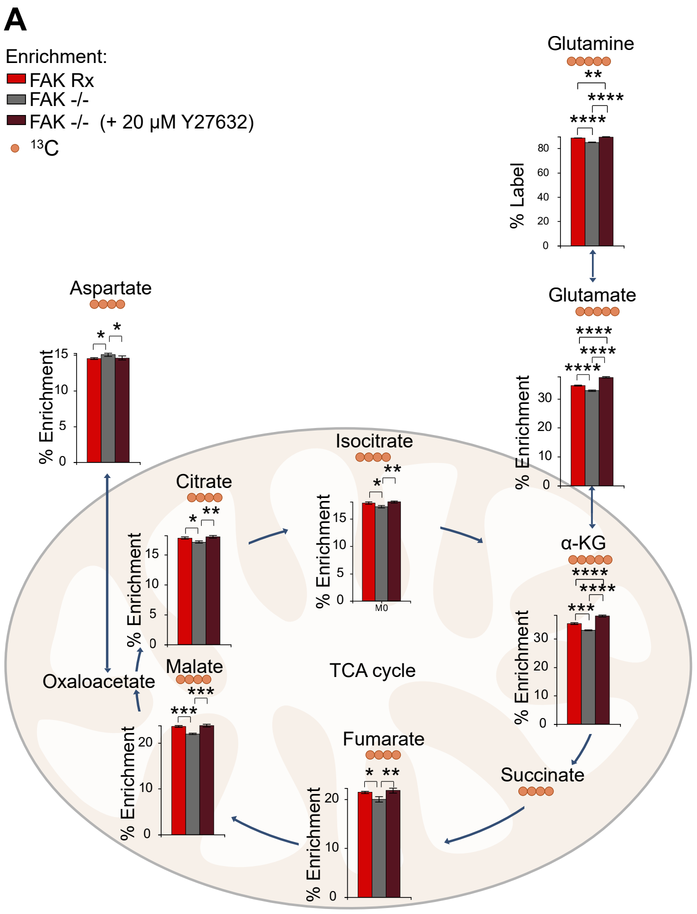

Fig. S5. Treating FAK -/- cells with ROCK inhibitor Y27632 increases glutamine oxidation.

(A) The atom fraction enrichment of glutamine-derived ^{13}C in TCA cycle intermediates in FAK Rx and FAK -/- cells treated with or without 20 µM Y27632 for 24 h followed by incubation for 3 h with $^{13}C_5$ glutamine supplemented medium. The main isotopologue of each metabolite is shown and plotted as the fraction of the sum of all isotopologues. Mean and SD are shown. Statistics: one-way ANOVA with a Tukey multiple test correction (n = 3 independent cultures on the same day).

Table S1. Reagents and resources.

Reagent or resource	Source	Identifier	Dilution
**Antibodies**			
Rabbit polyclonal anti-FAK	Cell Signaling Technology	Cat# 3285, RRID:AB_2269034	1:10000 for western blotting.
Rabbit monoclonal anti-COX IV	Cell Signaling Technology	Cat# 4850, RRID:AB_2085424	1:1000 for western blotting.
Rabbit polyclonal anti-Phospho-FAK (Tyr397)	Cell Signaling Technology	Cat# 3283, RRID:AB_2173659	1:1000 for western blotting.
Rabbit monoclonal anti-COFILIN	Cell Signaling Technology	Cat# 5175, RRID:AB_10622000	1:1000 for western blotting.
Rabbit monoclonal anti-Glut1	Cell Signaling Technology	Cat# 73015, RRID:AB_3064908	1:1000 for western blotting.
Rabbit monoclonal anti-Enolase-2	Cell Signaling Technology	Cat# 24330, RRID:AB_2868543	1:1000 for western blotting.
Mouse monoclonal Anti-Myosin Light Chain 2, phospho (Ser19)	Cell Signaling Technology	Cat# 3675, RRID:AB_2250969	1:200 for Immunofluorescence.
Rabbit monoclonal anti-Ki-67	Cell Signaling Technology	Cat# 12202, RRID:AB_2620142	1:1000 for immunohistochemistry
Rabbit polyclonal anti-NF1	Bethyl	Cat#A300-140A; RRID:AB_2149790	1:1000 for western blotting.
Rabbit monoclonal anti-PTEN	Cell Signalling Technology	Cat#9559; RRID:AB_390810	1:1000 for western blotting.
Rabbit monoclonal anti-EGFR	Cell Signalling Technology	Cat#4267; RRID:AB_2246311	1:1000 for western blotting.
Mouse monocolonal anti-α-Tubulin	Cell Signaling Technology	Cat#3873; RRID:AB_1904178	1:1000 for western blotting.
Rabbit polyclonal anti-GFP	ChromoTek	Cat# PABG1-20;RRID:AB_2749857	1:100 for immunohistochemistry
Alexa Fluor™ 568-conjugated anti-mouse IgG	Invitrogen	Cat# A-11004, RRID:AB_2534072	1:300 for Immunofluorescence.
**Bacterial and virus strains**			
One Shot™ Stbl3™ Chemically Competent *E. coli*	Invitrogen	Cat#C737303	
**Chemicals, peptides, and recombinant proteins**			
Laminin-I	R&D	Cat#3446-005-01	

Disease Models & Mechanisms • Supplementary information

	Systems	
Murine EGF	Peprotech	Cat#315-09
Human FGF-2	Peprotech	Cat#100-18b
MitoTracker™ Deep Red FM	Invitrogen	Cat#M22426
Phalloidin-Atto647N	Sigma-Aldrich	Cat#65906
GSK269962A	Cayman Chemical	Cat#19180
Y-27632 dihydrochloride	Tocris	Cat#1254
ProLong™ Glass Antifade Mountant	Invitrogen	Cat#P36980
Growth factor-reduced Matrigel®	Corning	Cat#354230
**Critical commercial assays**		
Lonza® Mouse Neural Stem Cell Nucleofector™ Kit	Lonza	Cat#VPG-1004
In-Fusion® HD Cloning Plus CE	TakaraBio	Cat#638916
Qubit RNA Broad Range assay kit	Thermo Scientific	Cat#Q10210
Qubit dsDNA HS assay kit	Thermo Scientific	Cat#Q32854
**Experimental models: Cell lines**		
NPE cells	Gift from Steven Pollard (Centre for Regenerative Medicine, The University of Edinburgh), described in Gangoso et al	N/A
NPE FAK-/- cells	This study	N/A
NPE FAK Rx cells	This study	N/A
**Experimental models: Organisms/strains**		
CD-1/Nude mouse	Charles River Laboratories	Strain Code 086
**Oligonucleotides**		
sg*ptk2*: 5'-GCAGTAGTGAGCCAACCACCT	This study	N/A

Disease Models & Mechanisms • Supplementary information

L_*Ptk2*_HD:aattgatccgcggccgcgccaccatggcagctgcttatcttgacc	This study	N/A
R_Ptk2_HD :gcggaattccggatcctcagtgtggccgtgtctg	This study	N/A
**Recombinant DNA**		
pSpCas9(BB)-2A-GFP (PX458)	a gift from Feng Zhang (Addgene plasmid # 48138 ; http://n2t.net/addgene:48138	RRID:Addgene_48138
pQCXIN-FAK	This study	N/A
**Software and algorithms**		
ImageJ v2.14.0/1.54f	ImageJ, RRID:SCR_003070	https://imagej.net/ij/
GraphPad Prism v9	GraphPad Prism (RRID:SCR_002798)	https://www.graphpad.com/
R v4.4.1	R Project for Statistical Computing, RRID:SCR_001905	https://www.r-project.org/
GSEA v4.3.1	Subramanian et al.	https://www.gsea-msigdb.org/gsea/index.jsp
IncuCyte Software v2020C	Sartorius	https://www.sartorius.com/
Skyline 21.2.0.568	Pino et al.	https://skyline.ms/wiki/home/software/Skyline/page.view?name=tutorial_hi_res_metabolomics

Disease Models & Mechanisms • Supplementary information
